# Regulation of chromatin dynamics by a calcium-dependent nucleoskeleton

**DOI:** 10.1016/j.jbc.2026.113104

**Published:** 2026-05-06

**Authors:** María José González, Michele Angela Rodrigues, Santo Diprima, Dejian Zhao, Thais Fernandes Bassani, Clara Couto Fernandez, Emma Kruglov, Michael H. Nathanson, Dawidson Assis Gomes

**Affiliations:** 1 Department of Biochemistry and Immunology, Universidade Federal de Minas Gerais (UFMG), Belo Horizonte, Minas Gerais, Brazil; 2 Section of Digestive Diseases, Internal Medicine, Yale University CT, New Haven, Connecticut, USA; 3 Center for Omics Sciences, IRCCS San Raffaele Scientific Institute, Milan, Italy; 4 Yale Center for Genome Analysis, Department of Genetics, Yale School of Medicine, New Haven, Connecticut, USA

**Keywords:** calcium, actin, MYH9, inositol 1,4,5-trisphosphate (IP3), ITPR, chromatin, nucleus

## Abstract

Growth factors selectively activate calcium signaling pathways in the cell nucleus, which in turn regulate gene transcription and other intranuclear events, but the specific way this is accomplished is not entirely understood. Growth factors increase inositol 1,4,5-trisphosphate (IP3) in the nucleus, which in turn releases calcium from intranuclear IP3 receptors, and the present study shows that this leads to transient assembly of an actin nucleoskeleton that associates with intranuclear nonmuscle myosin 2A (MYH9). Mass spectrometry suggests that much of the MYH9 cargo consists of components of the gene transcription machinery, and chromatin immunoprecipitation identified a number of specific genes that associate with the myosin in response to stimulation with growth factors. Together, these findings suggest that growth factors initiate gene transcription by transiently assembling an actin nucleoskeleton that works with MYH9 to bring specific genes to the transcription machinery.

The versatility of Ca^2+^ as a second messenger is fundamentally linked to its strict spatiotemporal distribution within the cell (1). Nuclear Ca^2+^ has been shown to specifically facilitate cellular processes that include gene transcription, cell proliferation, and tumor growth (2, 3). Nuclear Ca^2+^ signals can be regulated independently of their cytosolic counterparts (4), as the nucleus contains the necessary machinery for its own Ca^2+^ mobilization. This includes intranuclear phospholipase C (PLC) isoforms that locally hydrolyze phosphatidylinositol 4,5-bisphosphate to form IP3 (5), the nucleoplasmic reticulum (NR), which are invaginations of the nuclear envelope that contain intranuclear IP3 receptors (ITPRs) (6), which release Ca^2+^ into the nucleoplasm, and nuclear translocation of receptor tyrosine kinases (RTKs), which initiate local PLC activation (5, 7, 8, 9). However, the precise mechanisms that link local nuclear Ca^2+^ signaling to downstream nuclear architectural and mechanical changes remain largely unknown.

In parallel, nuclear actin has emerged as a fundamental component of nuclear architecture, participating in transcription, DNA repair, and chromatin mechanics (10, 11, 12). Dynamic polymerization of actin in the nucleus are highly regulated events essential for transcription, chromosome translocation, and related aspects of cellular adaptation to external stimuli (13, 14, 15). Moreover, G protein–coupled receptor activation can induce Ca^2+^ transients originating from the cytoplasm, which subsequently triggers the assembly of nuclear actin filaments, thereby influencing chromatin dynamics (16). However, whether and how signaling pathways that connect local nuclear signaling to nucleoskeletal reorganization remains a major gap in our understanding of nucleus biology.

To address this knowledge gap, the present study investigates the components and signaling cascades that transduce local nuclear IP3/Ca^2+^ signals to nuclear actin dynamics. In a previous article, it was found that the Ca^2+^ channel ITPR3 interacts with nonmuscle myosin IIA (MYH9) (17). Given the established ability of MYH9 to interact with actin (18), this finding led to the hypothesis that ITPR3 could drive the assembly of a nuclear actin nucleoskeleton. In this study, it is confirmed that ITPR3, MYH9, and β-actin localize to the nucleus and form a tripartite protein complex.

Evidence is provided that epidermal growth factor (EGF) stimulation increases nuclear IP3 (5), which releases Ca^2+^ from intranuclear ITPRs to trigger the transient assembly of this actin nucleoskeleton. By buffering nuclear IP3, it is demonstrated that this specific nuclear pool of IP3 is necessary for nuclear actin filament formation. To understand the functional consequence of this machinery on the genome, chromatin immunoprecipitation sequencing (ChIP-Seq) targeting MYH9 was employed. The results show that EGF stimulation promotes the association of MYH9 with specific genomic loci, an interaction that is dependent on nuclear IP3 signaling. Buffering nuclear IP3 results in a global reduction of MYH9 binding to chromatin, particularly at promoter regions and sites associated with cytoskeletal organization and chromatin remodeling. Collectively, these findings suggest that growth factors control gene transcription by transiently assembling an actin nucleoskeleton that works with MYH9 to bring specific genes to the transcription machinery. This establishes a novel paradigm where RTK-mediated signaling acts as a trigger for nuclear IP3 to orchestrate nuclear actin filament formation and chromatin organization.

## Results

### ITPR3 forms a complex in the nucleus with MYH9 and β-actin

**Figure 1:**
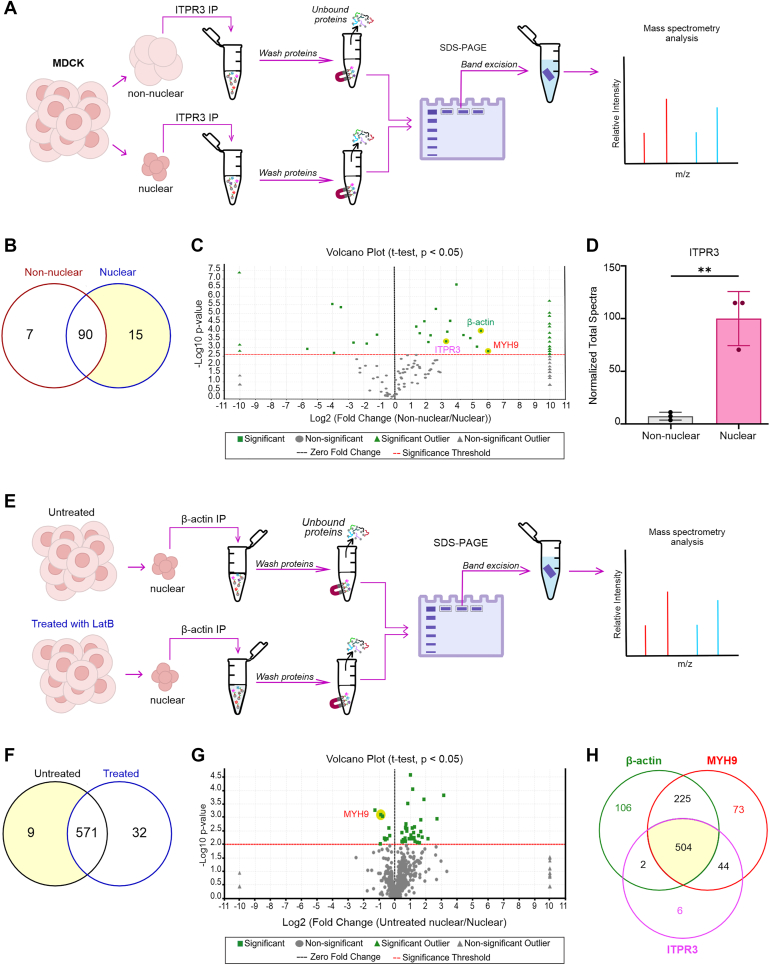
**ITPR3 forms a complex in the nucleus with MYH9 and β-actin.***A,* schematic representation of the proteomic workflow in MDCK cells, which involves subcellular fractionation into nuclear and non-nuclear compartments followed by immunoprecipitation (IP) of ITPR3. *B*, Venn diagram analysis identified 15 proteins differentially expressed in the nuclear fraction, including ITPR3. *C*, volcano plot highlighting key proteins with significant differential expression in the nuclear fraction following ITPR3 IP. *D*, ITPR3 is highly expressed in the nuclear fraction of MDCK cells. Data are presented as mean ± SD; ∗∗*p* < 0.01, the *p* value was obtained by unpaired two-tailed *t* test. *E,* schematic representation of the proteome workflow shows U2OS cells treated or untreated with 200 nM of latrunculin B (LatB) for 12 h, followed by β-actin IP from nuclear fractions. *F*, the Venn diagram reveals that nine proteins are more highly expressed in untreated cells, where actin filaments remain preserved. *G*, volcano plot showing that MYH9 is among the most significantly differentially expressed proteins when actin filaments are intact. *H,* the Venn diagram illustrates that the majority of the ITPR3 interactome is shared with β-actin and MYH9 interactors. n = 3 from each group.

The ITPR3 nuclear interactome in MDCK cells was characterized by proteomic analysis by comparing ITPR3 immunoprecipitates from the nuclear and non-nuclear fractions (Fig. 1*A*). Differential expression analysis of the ITPR3 interactome using Scaffold software revealed 15 proteins in the nuclear fraction, as shown in the Venn diagram (Fig. 1*B*). Among the 15 proteins identified in the nuclear fraction, MYH9 and β-actin were found to interact with ITPR3 (Fig. 1*C* and Table 1). As ITPR3 is found enriched within the nucleus in these cells (Fig. 1*D*), we hypothesized that ITPR3 could release Ca^2+^ within the nucleus and form a complex with MYH9 to regulate the assembly of nuclear actin filaments. To investigate whether the interaction of MYH9 with β-actin is dependent on intact F-actin structures, a proteomic assay was performed using U2OS cells, which facilitate the visualization of nuclear actin filaments, to select proteins that could participate in nuclear actin assembly. Cells were treated with latrunculin B (Lat-B) to inhibit actin filament formation to identify protein interactions with nuclear actin filaments (Fig. 1*E*). Differential analysis of the β-actin interactome in nuclear fractions revealed nine proteins in untreated nuclei (where filaments were intact), 32 proteins were identified in Lat-B-treated nuclei. MYH9 was among the nine proteins detected in the untreated nuclei (Fig. 1, *F* and *G* and Table 2). Further support for this interaction was provided by analysis using the STRING database, which confirmed the presence of the same nine proteins identified in Figure 1*F* and revealed potential functional associations among them (Fig. S1). Next, the interactome of ITPR3, MYH9, and β-actin was compared. The Venn diagram shown in Figure 1*H* (Table 3) illustrates that 90.7% of the ITPR3 interactome is shared with β-actin and MYH9 interactors. This result suggests that ITPR3 may form a complex with this actin-myosin network.

**Table 1:** Most highly expressed nuclear proteins interacting with ITPR3 in MDCK cells

Access number	Protein name	Molecular weight kDa	*t* test (*p* value)
E2RMC0	Inositol 1,4,5-trisphosphate receptor type 3	304	<0.00010
F1PLS4	Vimentin	58	0.081
O18840	ACTB	24	0.073
F1PLV2	Peptidyl-prolyl cis-trans isomerase	25	0.00039
F1PHS5	Plectin	534	0.00092
J9JH91	Envoplakin	64	0.066
F1Q0H6	Inositol 1,4,5-trisphosphate receptor type 2	234	0.0016
J9NU37	Periplakin	308	0.002
E2QW54	LIM domain and actin binding	204	<0.00010
Q6TEQ7	Annexin	55	0.0002
F1PIQ7	Inositol 1,4,5-trisphosphate receptor type 1	281	0.0097
E2R0F2	Filamin B	130	0.011
F1PNH8	ITPR interacting domain containing 2	77	<0.00010
J9NSL9	Sciellin	21	0.025
F1P9J3	Myosin-9	226	0.04

**Table 2:** Most highly expressed nuclear proteins interacting with F-actin in U2OS cells

Access number	Protein name	Molecular weight kDa	*t* test (*p* value)
Q09666	Neuroblast differentiation-associated protein AHNAK	629	0.00054
P35579	Myosin-9	227	0.0067
P21333	Filamin-A	281	0.0024
P61978	Heterogeneous nuclear ribonucleoprotein K	51	0.0047
P35580	Myosin-10	229	0.00089
O75369	Filamin-B	278	0.0061
Q9UHB6	LIM domain and actin-binding protein 1	85	0.006
Q05682	Caldesmon	93	0.0008
P02452	Collagen alpha-1 (I) chain	139	0.0094

**Table 3:** Common interactors of β-actin, MYH9, and ITPR3

Access number	Protein name	Molecular weight kDa
Q15149	Plectin	532
P60709	ACTB	42
P08670	Vimentin	54
Q09666	Neuroblast differentiation-associated protein AHNAK	629
Q562R1	Beta-actin-like protein 2	42
P23284	Peptidyl-prolyl cis-trans isomerase B	24
P21333	Filamin-A	281
P35579	Myosin-9	227
P78527	DNA-dependent protein kinase catalytic subunit	469
P52272	Heterogeneous nuclear ribonucleoprotein M	78
P09651	Heterogeneous nuclear ribonucleoprotein A1	39
Q08211	ATP-dependent RNA helicase A	141
P07910	Heterogeneous nuclear ribonucleoproteins C1/C2	34
P22626	Heterogeneous nuclear ribonucleoproteins A2/B1	37
P07355	Annexin A2	39
P43243	Matrin-3	95
Q01082	Spectrin beta chain, nonerythrocytic 1	275
Q13813	Spectrin alpha chain, nonerythrocytic 1	285
Q00839	Heterogeneous nuclear ribonucleoprotein U	91
P61978	Heterogeneous nuclear ribonucleoprotein K	51
P26599	Polypyrimidine tract-binding protein 1	57
P38159	RNA-binding motif protein, X chromosome	42
Q6P2Q9	Pre-mRNA–processing-splicing factor 8	274
P17844	Probable ATP-dependent RNA helicase DDX5	69
P02545	Prelamin-A/C	74
O75369	Filamin-B	278
P12814	Alpha-actinin-1	103
P0C0S5	Histone H2A.Z	14
Q12906	Interleukin enhancer-binding factor 3	95
P39019	Small ribosomal subunit protein eS19	16
P23396	Small ribosomal subunit protein uS3	27
P35580	Myosin-10	229
P20700	Lamin-B1	66

### ITPR3, MYH9, and β-actin are localized to the nucleus

**Figure 2:**
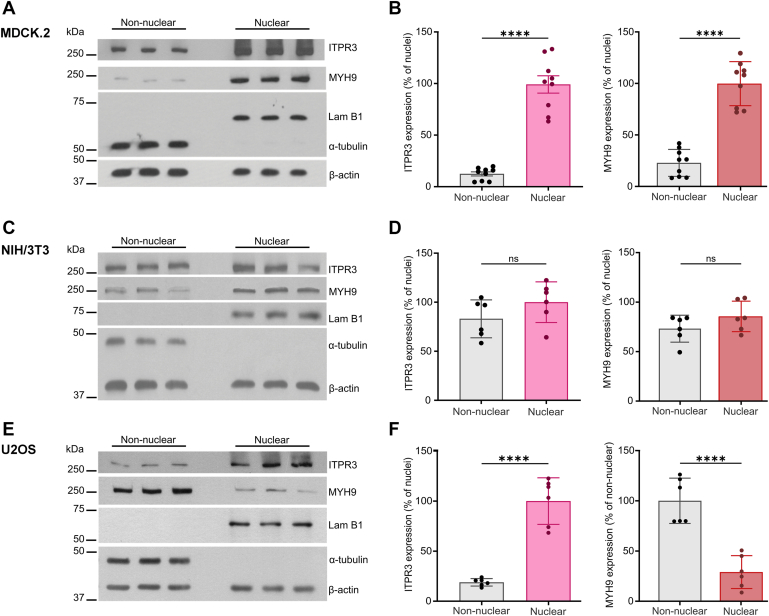
**ITPR3, MYH9, and β-actin are found within the nucleus.***A,* Western blot analysis of non-nuclear and nuclear ITPR3, MYH9, and β-actin in MDCK cells. *B,* quantification of Western blot data indicates that ITPR3 and MYH9 are enriched in the nuclear fraction of MDCK cells (*p* < 0.0001; n = 9 per group). *C,* Western blot analysis of non-nuclear and nuclear ITPR3, MYH9, and β-actin in NIH/3T3 cells. *D*, bar graphs derived from Western blot analysis show similar expression levels of ITPR3 and MYH9 in nuclear and non-nuclear fractions (n = 6 per group). *E*, Western blot analysis of non-nuclear and nuclear ITPR3, MYH9, and β-actin in U2OS WT cells. *F*, graphs demonstrate that ITPR3 is significantly expressed in the nuclear fraction, while MYH9 shows reduced nuclear expression (*p* < 0.0001; n = 6 per group). α-tubulin and lamin B1 (LamB1) were used as purity controls for non-nuclear and nuclear fractions, respectively and β-actin was used as the loading control. Indicated kDa values represent the position of the referenced bands from prestained protein standard. Data are presented as mean ± SD. The *p* values were obtained by unpaired two-tailed *t* test (∗∗∗∗*p* < 0.0001, ns = not significant). Experiments were performed in triplicate (n = 3 independent experiments). Scale bars represent 5 μm.

Proteomic analysis demonstrated that ITPR3, MYH9, and β-actin are all present in the nucleus. To validate these findings, Western blotting was performed on MDCK, NIH/3T3, and U2OS cells following cellular fractionation, using Lamin B1 and α-tubulin as markers for the nuclear and cytoplasmic compartments, respectively. In MDCK cells, both ITPR3 and MYH9 were predominantly present in the nuclear fraction, with enrichment levels of 86.7 ± 8.7% for ITPR3 and 77.0 ± 8.4% for MYH9 (Fig. 2, *A* and *B*). In NIH/3T3 cells, the levels of these proteins were comparable between the nuclear and cytosolic fractions (Fig. 2, *C* and *D*). In U2OS cells, nuclear ITPR3 was higher than its cytosolic counterpart, with a difference of 81.1 ± 9.6%, while nuclear MYH9 was lower than cytosolic MYH9, showing a difference of 71.0 ± 11.4% (Fig. 2, *E* and *F*). Collectively, these findings demonstrate that ITPR3, MYH9, and β-actin are expressed in the nucleus.

### Calcium induces the formation of nuclear actin filaments

Previous studies have demonstrated that stimulation with fetal bovine serum (FBS) or the Ca^2+^ ionophore ionomycin can induce the formation of nuclear actin filaments in NIH/3T3 cells (16). To further examine the effects of these stimuli on Ca^2+^ dynamics, both U2OS and NIH/3T3 cells were treated with FBS or ionomycin. U2OS and NIH/3T3 cells were first cultured in FBS-free Dulbecco’s modfied Eagle’s medium (DMEM) for 12 h and then incubated with the Ca^2+^ dye Fluo-4 AM. Stimulation with FBS led to elevated Ca^2+^ levels in both the nucleus and cytosol of U2OS and NIH/3T3 cells (Fig. S2, *A–C* and *G–I*). Comparable results were obtained when ionomycin was used as a stimulus (Fig. S2, *D–F* and *J–L*), confirming that Ca^2+^ can be detected in both the nucleus and cytosol under these conditions.

**Figure 3:**
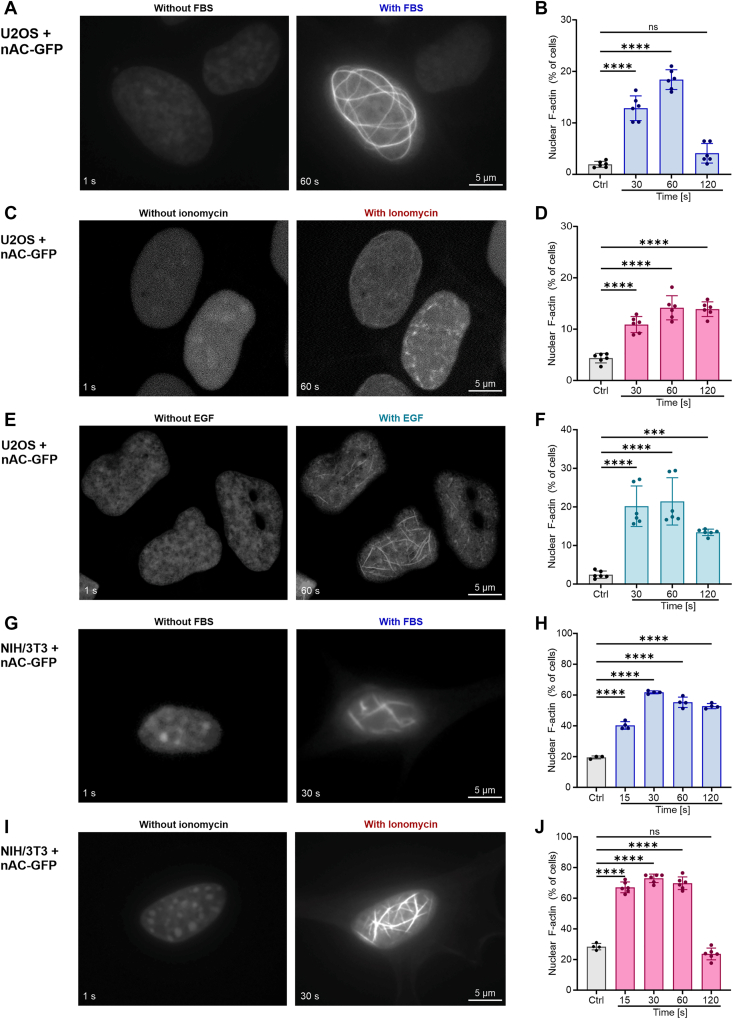
**Agents that increase calcium in the nucleus induce the assembly of nuclear actin filaments.***A*, representative images of U2OS cells stably expressing nAC–GFP, shown before (*left*) and after stimulation with 20% FBS (*right*). *B*, the graph illustrates a time-dependent increase in nuclear actin filament formation at 30, 60, and 120 s after stimulation with 20% FBS by 10.89  ±  1%, 16.45  ±  1%, and 2.15  ±  1%, respectively. *C*, representative images of U2OS cells stably expressing nAC–GFP, shown before (*left*) and after stimulation with 2 μM of ionomycin (*right*). *D*, the graph shows an increase in nuclear F-actin formation at 30, 60, and 120 s after stimulation with ionomycin by 6.5  ± 0.1%, 9.8  ±  0.1%, and 9.5  ±  1%, respectively. *E*, representative images of U2OS cells stably expressing nAC–GFP, shown before (*left*) and after stimulation with 200 ng/ml EGF (*right*). *F*, the graph shows an increase in nuclear F-actin formation at 30, 60, and 120 s after stimulation with EGF by 18  ±  2%, 19  ±  2%, and 11 ±  2%, respectively. *G*, representative images of NIH/3T3 cells stably expressing nAC–GFP, shown before (*left*) and after stimulation with 20% FBS (*right*). *H*, the graph illustrates a time-dependent increase in nuclear actin filament formation at 15, 30, 60, and 120 s after stimulation with 20% FBS by 21  ±  2%, 42  ±  2%, 36  ±  2%, and 33  ±  2%, respectively. *I*, representative images of NIH/3T3 cells stably expressing nAC–GFP, shown before (*left*) and after stimulation with 2 μM of ionomycin (*right*). *J*, the graph shows an increase in nuclear F-actin formation at 15, 30, 60, and 120 s after stimulation with ionomycin by 39 ± 2%, 45  ±  2%, 41  ±  2%, and 5  ±  2% respectively. For each experiment with U2OS WT, ∼350 cells were recorded and counted per replicate, and for NIH/3T3, ∼200 cells were recorded and counted per replicate. Experiments were performed in triplicate (n = 3 independent experiments). Scale bars represent 5 μm. Images were acquired using Leica GSD/TIRF HP or Leica STED microscopes. Statistical analysis was performed using two-way ANOVA test with Dunnet correction (∗∗∗∗*p* ≤ 0.0001, ns, not significant). Data are mean ± SD. All experiments were performed at least three times independently.

Based on these results, actin dynamics and the possible mechanisms underlying filament formation were further investigated. To visualize nuclear actin, cells were transfected with the “nuclear actin chromobody” (nAC), a nanobody against actin fused to a GFP and carrying a nuclear localization signal (NLS), which enabled monitoring of filament assembly specifically in the nucleus. U2OS cells expressing the nAC construct were first monitored after stimulation with FBS (Fig. 3*A*) or ionomycin (Fig. 3*C*, Video S1). These findings were quantified in fixed cells, where the peak formation of nuclear actin filaments occurred 60 s after FBS stimulation (Fig. 3*B*). Stimulation with the Ca^2+^ ionophore also induced nuclear actin filament assembly (Fig. 3*C*), with a peak at 60 s (Fig. 3*D*). To validate these findings in an additional cell line, this experiment was repeated in NIH/3T3 cells, as previously demonstrated by others (16). Both agonists also triggered nuclear actin filament formation in these cells, with a peak at 30 s (Fig. 3, *G–J*). Epidermal growth factor (EGF) increases nuclear IP3 and Ca^2+^ levels within the nucleus (5), so the effects of this agonist on nuclear actin filament assembly was examined in U2OS nAC cells. This treatment also resulted in the formation of nuclear actin filaments, with the assembly reaching its maximum at 60 s (Fig. 3, *E* and *F*). These results confirm that nuclear actin filament formation is a rapid response to stimulation with FBS and EGF.

### ITPR3 and nuclear IP3 transients are required for the assembly and regulation of the nuclear MYH9–β-actin complex

**Figure 4:**
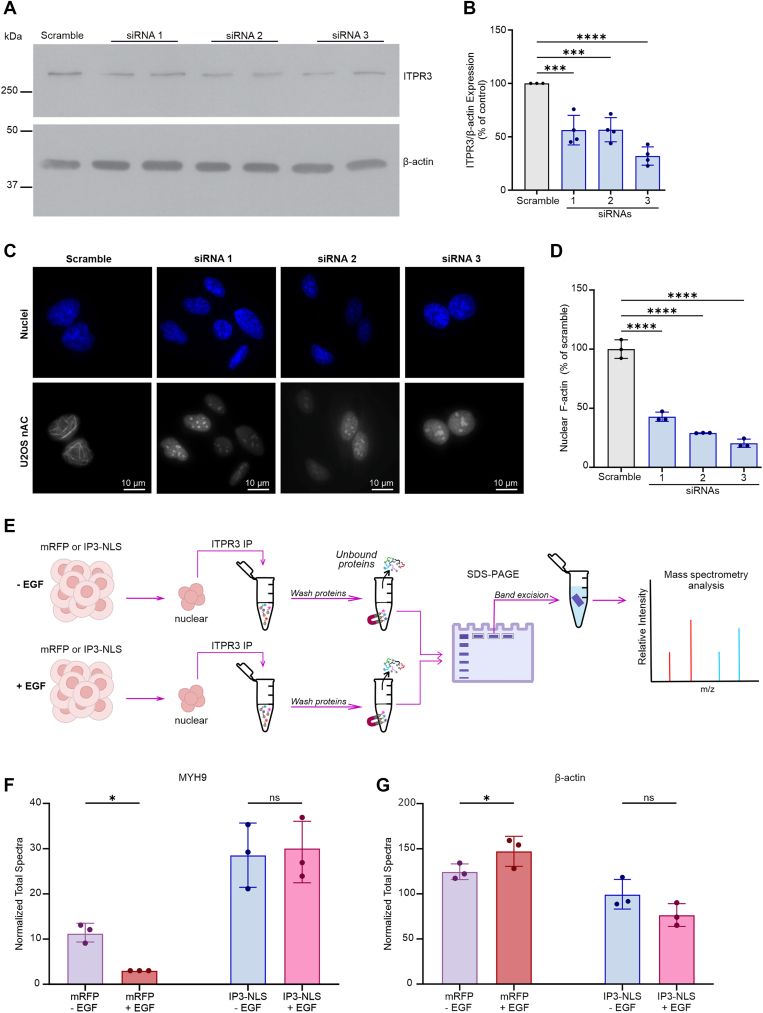
**The formation of nuclear F-actin depends on ITPR3.***A*, representative Western blot showing the silencing of ITPR3 in U2OS cells transfected with three different siRNA sequences. *B*, quantification of ITPR3 expression after silencing with siRNAs. Scrambled siRNA was used as a control. ITPR3 expression was significantly reduced by siRNA1 (44 ± 8%), siRNA2 (43 ± 8%), and siRNA3 (68 ± 8%). Data are means ± SD (n = 3 independent experiments). Statistical analysis was performed using a two-way ANOVA test with Dunnet correction (∗∗∗∗*p* ≤ 0.0001, ∗∗∗*p* ≤ 0.001). *C*, representative images of U2OS cells expressing nAC–GFP, showing a reduction in nuclear F-actin formation after silencing with scrambled, siRNA1, siRNA2, and siRNA3. *D*, the graph shows the percentage of cells with nuclear F-actin after silencing ITPR3 and stimulated with 20% FBS. The percentage of cells with nuclear F-actin was significantly reduced after silencing ITPR3 with siRNA1 (57 ± 4%), siRNA2 (71 ± 4%), and siRNA3 (80 ± 4%) compared to scrambled. For each experiment, the percentage of cells positive for nuclear F-actin was determined by analyzing approximately 250 cells per replicate. Data represents three independent biological experiments. Statistical analysis was performed using a two-way ANOVA test with Dunnet correction (∗∗∗∗*p* ≤ 0.0001). Scale bar represents 10 μm. *E*, schematic representation of the proteome analysis of nuclear fractions from cells infected with adenovirus expressing mRFP or IP3-NLS and stimulated or not with EGF. *F*, normalized total spectra of MYH9 associated with ITPR3. EGF stimulation led to a decrease in the interaction between MYH9 and ITPR3. In contrast, the expression of a nuclear-targeted IP3-NLS buffer abolished this EGF-induced change, maintaining the interaction at levels comparable to nonstimulated control. *G*, normalized total spectra of β-actin associated with ITPR3. The interaction between ITPR3 and β-actin increased following EGF stimulation. However, this interaction remained unchanged in cells expressing the nuclear IP3-NLS buffer. The *p* value was obtained by unpaired two-tailed *t* test, ∗*p* ≤ 0.05 (n = 3). Data are means ± SD.

ITPRs function as intracellular as well as intranuclear Ca^2+^ release channels (1, 6, 19). Because EGF stimulated filament assembly and ITPR3 is in the nucleus of these cells, we investigated whether the nuclear ITPR channel is responsible for the growth factor–induced assembly of nuclear actin filaments. To investigate this, ITPR3 was knocked down in U2OS cells using three siRNA sequences (Table 4). Western blot analysis showed knockdown efficiencies of 44 ± 8%, 43 ± 8%, and 68 ± 8% for sequences 1 to 3, respectively, compared with scramble control (Fig. 4, *A* and *B*). Quantification of nuclear F-actin in U2OS nAC cells upon FBS stimulation at the established peak time of 60 s (Fig. 4*C*) showed that actin filament assembly was reduced by 57.2 ± 4%, 71 ± 4%, and 80 ± 4% for sequences 1 to 3, respectively, relative to scramble controls (Fig. 4*D*). To corroborate these findings, ITPR3 was knocked down in NIH/3T3 cells using siRNA3, which had the highest knockdown efficiency (Fig. S3*A*). Western blot analysis confirmed a 96.9 ± 5% reduction relative to scramble controls (Fig. S3*B*). Quantification in NIH/3T3 nAC cells further revealed that FBS stimulation followed by fixation at 60 s (Fig. S3*C*) resulted in a 64 ± 2% reduction in nuclear actin filament assembly compared with scramble controls (Fig. S3*D*). These results are consistent with the idea that Ca^2+^ release *via* ITPR3 participates in nuclear actin assembly.

**Table 4:** Sequences of ITPR3 siRNAs used in this study

Name	Sequence	Cat. number
siRNA 1	5′-GGACUGACAAGAAUAACGAGCAUCA-3	442784242
siRNA 2	5′-GACAUCAUGGUCACUAAGCCCAACC-3′	442784245
siRNA 3	5′-CAGUUUACCUUAAUGCCUUAGCAGA-3′	442784248
Scramble	(DsiRNA) 5 nmol, negative control	51-01-14-04

Next, the role of EGF-induced nuclear IP3 signaling in forming interactions between ITPR3, MYH9, and β-actin was investigated. U2OS cells were infected with an adenoviral construct encoding a monomeric red fluorescent protein (mRFP) tagged with the IP3 binding domain of ITPR1, along with a NLS (IP3-NLS), which selectively buffers nuclear IP3 (8). This approach enabled assessment of the effect of EGF-induced nuclear IP3 signaling on the assembly and regulation of the ITPR3–MYH9–β-actin complex. Experiments were performed with cells expressing either mRFP alone or IP3-NLS, followed by EGF stimulation or no stimulation. Nuclear fractions were isolated, and immunoprecipitation was performed using an ITPR3-specific antibody (Fig. 4*E*). Notably, EGF stimulation led to a decreased association of MYH9 with ITPR3 (Fig. 4*F*), while the interaction between β-actin and ITPR3 increased compared to controls (Fig. 4*G*). By contrast, in cells expressing the IP3-NLS buffer, these EGF-induced changes were not observed, and the interactions remained comparable to nonstimulated cells (Fig. 4, *F–G*). Immunofluorescence analysis revealed that EGF treatment promoted nuclear contact points between MYH9 and β-actin (Fig. S4, *A* and *B*), while diminishing MYH9–ITPR3 interactions (Fig. S4, *C* and *D*). These findings show that EGF-mediated nuclear IP3 signaling dynamically regulates the ITPR3–MYH9–β-actin complex within the nucleus.

### Nuclear IP3 is necessary for nuclear actin filament formation

**Figure 5:**
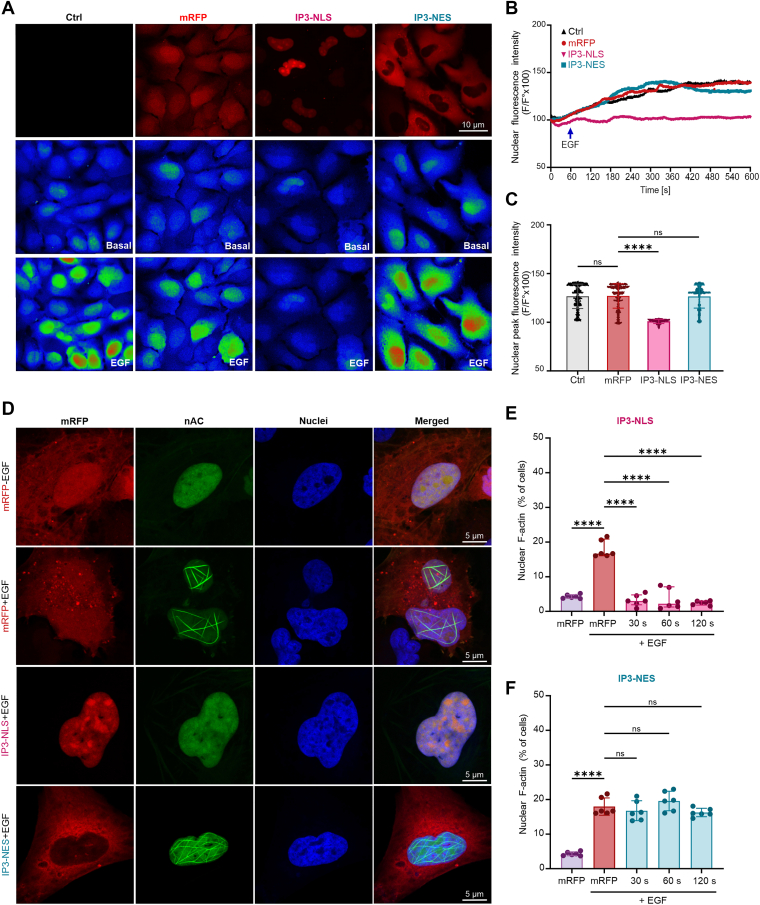
**Nuclear IP3 is necessary for nuclear actin filament formation.***A*, representative images showing the localization of IP3-NLS and IP3-NES buffers in U2OS cells. Images were taken before (Basal) and after stimulation with 200 ng/ml of EGF. Scale bars represent 10 μm. *B*, graph showing calcium dynamics in U2OS cells after stimulation with EGF (at t = 60 s) for uninfected (Ctrl) cells and those expressing mRFP, IP3-NES, or IP3-NLS. *C*, quantification of nuclear peak calcium intensity demonstrates that IP3-NLS decreases the nuclear calcium peak after EGF stimulation, while IP3-NES does not. *D*, representative images of U2OS cells stably expressing nAC–GFP after being infected with adenovirus to express mRFP, IP3-NLS, or IP3-NES buffers and stimulated with 200 ng/ml of EGF. Scale bars represent 5 μm. *E*, buffering IP in the nucleus, with IP3-NLS, blocked EGF-induced formation of actin filaments in the nucleus. *F*, buffering IP in the cytosol does not reduce EGF-induced formation of actin filaments in the nucleus. mRFP – EGF was used as a negative control and mRFP + EGF as a positive control. For each experiment, the percentage of cells positive for nuclear F-actin was determined by analyzing approximately 350 cells per replicate. Data represents six independent biological experiments. Statistical analysis was performed using a two-way ANOVA test with Dunnet correction (∗∗∗∗*p* ≤ 0.0001, ns = not significant). Data are means ± SD. Images were acquired using a Leica-gated STED super-resolution microscope.

ITPR3 knockdown suggested that this receptor is required for the Ca^2+^ release necessary for actin filament assembly. Since ITPR3 is present in the nuclear fraction, it was hypothesized that nuclear IP3 signaling is necessary for nuclear actin assembly. To investigate this, Ca^2+^ assays were performed in U2OS cells infected with mRFP alone, IP3-NLS, or IP3 buffer with a nuclear exclusion signal (IP3-NES), alongside uninfected cells as a negative control (Ctrl). Cells were cultured in DMEM without FBS for 12 h, loaded with Fluo-4 AM, and stimulated with 200 ng/ml of EGF. The cells were then transferred to a perfusion chamber, and Ca^2+^ dynamics were monitored in both the cytoplasm and nucleus (Fig. 5*A*). EGF increased Ca^2+^ levels in the nucleus and cytoplasm of cells infected with IP3-NES, mRFP, or uninfected cells (Ctrl). In contrast, no increase in Ca^2+^ was detected in either compartment in cells infected with IP3-NLS (Fig. 5*B*). Next, we quantified the peak intensity of the Ca^2+^ signals and found no differences between cells infected with IP3-NES, mRFP, and the uninfected control (Fig. 5*C*). In contrast, cells infected with IP3-NLS failed to respond to EGF stimulation (Fig. 5, *B* and *C*).

Because IP3-NLS reduced intracellular Ca^2+^ release, we investigated whether nuclear IP3 is directly required for nuclear actin assembly. To address this, the formation of nuclear actin filaments was quantified in U2OS cells expressing the nAC construct after infection with IP3-NLS, IP3-NES, or mRFP adenoviruses. Cells were cultured in DMEM without FBS for 12 h and then stimulated with 200 ng/ml EGF for 30, 60, or 120 s. Two controls were included: mRFP-infected cells stimulated with EGF (positive control, Ctrl +EGF) and unstimulated mRFP-infected cells (negative control, Ctrl -EGF) (Fig. 5*D*). The results showed that cells infected with IP3-NLS buffer and stimulated with EGF failed to form nuclear actin filaments (Fig. 5*E*). In contrast, cells infected with IP3-NES formed nuclear actin filaments upon stimulation, with no difference compared with the stimulated control (Fig. 5*F*). We also found that nuclear IP3 mediates nuclear actin filament assembly in response to 20% FBS (Fig. S5, *A–C*). These results indicate that both EGF and FBS stimulate Ca^2+^ release *via* nuclear IP3 signaling, which is necessary for nuclear actin filament assembly in these cells.

### Nuclear IP3 blocks EGF-induced changes in ITPR3-associated proteins involved in RNA metabolism

**Figure 6:**
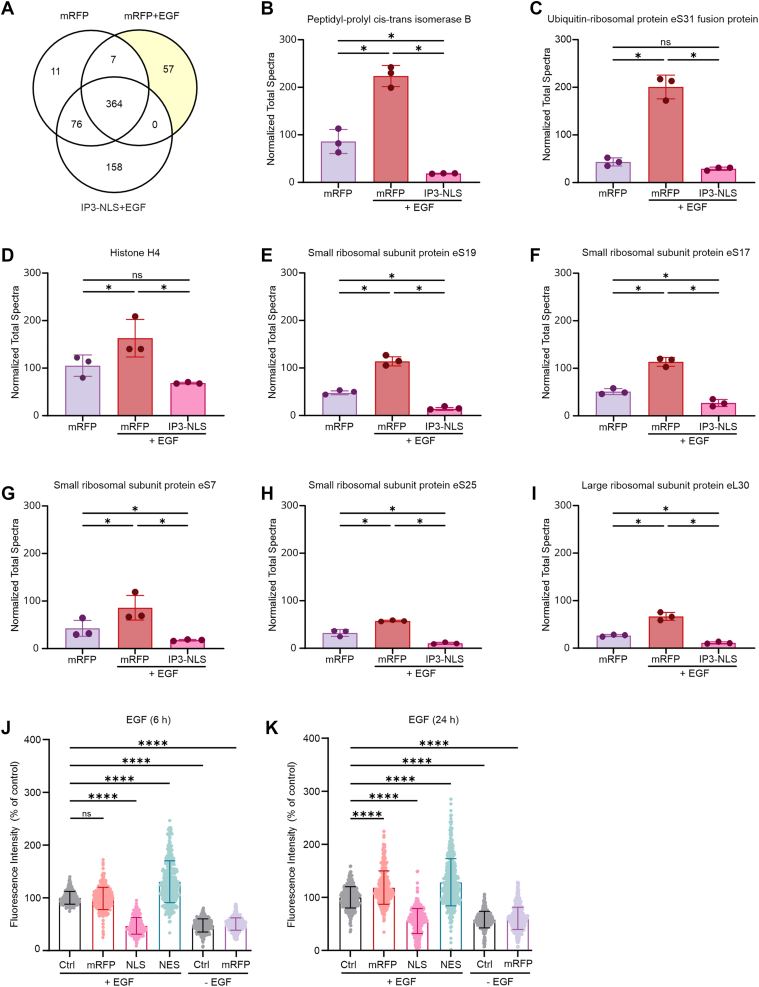
**Buffering IP3 in the nucleus blocks EGF-induced changes in ITPR3-associated proteins involved in RNA metabolism.***A*, Venn diagram showing the overlap of proteins identified by mass spectrometry in U2OS cells treated with mRFP control, mRFP + EGF, and IP3-NLS + EGF. The *yellow-shaded* area represents the 57 proteins found exclusively in the mRFP + EGF group, whose association with ITPR3 was blocked by IP3-NLS treatment. *B–I*, normalized total spectra of representative proteins from the mRFP, mRFP + EGF, and IP3-NLS + EGF treatment groups. These include peptidyl-prolyl cis-trans isomerase B (*B*), ubiquitin ribosomal protein ES31 fusion protein (*C*), histone H4 (*D*), small ribosomal subunit protein eS19 (*E*), small ribosomal subunit protein eS17 (*F*), small ribosomal subunit protein eS7 (*G*), small ribosomal subunit protein eS25 (*H*), large ribosomal subunit protein eL30 (*I*). Data are presented as mean ± SD; ∗*p* < 0.05, One-way ANOVA corrected for multiple comparisons (Benjamini, Krieger, and Yekutieli method); (n = 3). The minimum value for lower-expressed proteins was three peptides. *J* and *K*, nuclear IP3 buffering reduces total RNA synthesis after 6 h (by 53.1%) and 24 h (by 44.7%). Statistical analysis was performed using a two-way ANOVA test with Dunnet correction (∗*p* < 0.05 and ∗∗∗∗*p* < 0.0001). Data are means ± SD.

To investigate the role of nuclear IP3 in EGF-induced reorganization of the ITPR3 nuclear interactome, nuclear IP3 was buffered (Fig. 6). Proteomic analysis of nuclei revealed a population of 57 proteins whose interaction with ITPR3 increased following EGF stimulation and was subsequently blocked in the presence of the IP3-NLS construct (Fig. 6*A* and Table 5), many of which are linked to RNA metabolism (Fig. 6, *B–I*). This group represents proteins whose increased interaction with ITPR3 is directly dependent on EGF-mediated nuclear IP3 signaling. Furthermore, nuclear proteomic analysis showed a group of 76 proteins whose interaction with ITPR3 decreased following EGF stimulation and was subsequently blocked by the IP3-NLS construct, many of which are also associated with RNA metabolism (Fig. S6*A* and Table 6). To functionally characterize these ITPR3-associated proteins, pathway enrichment analysis was performed using Reactome for the 57 proteins whose interaction with ITPR3 increased following EGF stimulation and was subsequently blocked in the presence of the IP3-NLS construct. The majority of these proteins are involved in mRNA metabolism (Fig. 6, *B*–*I*). Further deconvolution of enriched processes revealed specific associations with pathways such as mRNA capping, nonsense-mediated decay, and processing of capped intron-containing pre-mRNA. These findings suggest that EGF stimulation, *via* ITPR3 and nuclear IP3 release, dynamically recruits key components of the mRNA processing and decay machinery to the ITPR3-associated nucleoskeleton complex. To confirm the functional significance of these observations, the impact of nuclear IP3 buffering on global RNA synthesis was evaluated. Detection of nascent RNA by 5-ethynyl uridine (Click-EU) labeling showed that the nuclear IP3 buffer reduces total RNA synthesis by 53.1 ± 1.6% in 6 h (Fig. 6*J*) and by 44.7 ± 2% in 24 h (Fig. 6*K*). This observation provides evidence for the functional importance of the mRNA metabolism-related proteins that become associated with intranuclear ITPR3 upon stimulation with EGF.

**Table 5:** Protein list found exclusively in the mRFP + EGF group

Accession number	Protein name	Molecular weight kDa	*p* value
P62805	Histone H4	11	<0.05
P60709	ACTB	42	<0.05
O60814	Histone H2B type 1-K	14	<0.05
P06899	Histone H2B type 1-J	14	<0.05
P62979	Ubiquitin-ribosomal protein eS31 fusion protein	18	<0.05
P23284	Peptidyl-prolyl cis-trans isomerase B	24	<0.05
P07355	Annexin A2	39	<0.05
P68104	Elongation factor 1-alpha 1	50	<0.05
P61247	Small ribosomal subunit protein eS1	30	<0.05
P08708	Small ribosomal subunit protein eS17	16	<0.05
Q14573	Inositol 1,4,5-trisphosphate receptor type 3	304	<0.05
Q9P2E9	Ribosome-binding protein 1	152	<0.05
P62701	Small ribosomal subunit protein eS4, X isoform	30	<0.05
P16403	Histone H1.2	21	<0.05
Q9H0A0	RNA cytidine acetyltransferase	116	<0.05
P26583	High mobility group protein B2	24	<0.05
P10412	Histone H1.4	22	<0.05
P62081	Small ribosomal subunit protein eS7	22	<0.05
P39019	Small ribosomal subunit protein eS19	16	<0.05
Q05639	Elongation factor 1-alpha 2	50	<0.05
P62906	Large ribosomal subunit protein uL1	25	<0.05
Q9Y295	Developmentally regulated GTP-binding protein 1	41	<0.05
P27816	Microtubule-associated protein 4	121	<0.05
P62851	Small ribosomal subunit protein eS25	14	<0.05
P62888	Large ribosomal subunit protein eL30	13	<0.05
Q9H0S4	Probable ATP-dependent RNA helicase DDX47	51	<0.05
Q00688	Peptidyl-prolyl cis-trans isomerase FKBP3	25	<0.05
Q9UBU8	Mortality factor 4-like protein 1	41	<0.05
P09429	High mobility group protein B1	25	<0.05
P62316	Small nuclear ribonucleoprotein Sm D2	14	<0.05
Q96AG4	Leucine-rich repeat-containing protein 59	35	<0.05
Q9Y2W2	WW domain-binding protein 11	70	<0.05
P39748	Flap endonuclease 1	43	<0.05
Q14571	Inositol 1,4,5-trisphosphate receptor type 2	308	<0.05
P27695	DNA repair nuclease/redox regulator APEX1	36	<0.05
P37108	Signal recognition particle 14 kDa protein	15	<0.05
P55145	Mesencephalic astrocyte-derived neurotrophic factor	21	<0.05
O14776	Transcription elongation regulator 1	124	<0.05
Q8WU90	Zinc finger CCCH domain-containing protein 15	49	<0.05
P46821	Microtubule-associated protein 1B	271	<0.05
Q13268	Dehydrogenase/reductase SDR family member 2, mitochondrial	30	<0.05
Q9NV06	DDB1- and CUL4-associated factor 13	51	<0.05
Q00059	Transcription factor A, mitochondrial	29	<0.05
Q14554	Protein disulfide-isomerase A5	60	<0.05
P08238	Heat shock protein HSP 90-beta	83	<0.05
P47813	Eukaryotic translation initiation factor 1A, X-chromosomal	16	<0.05
P28290	Protein ITPRID2	138	<0.05
O15347	High mobility group protein B3	23	<0.05
O00566	U3 small nucleolar ribonucleoprotein protein MPP10	79	<0.05
P20042	Eukaryotic translation initiation factor 2 subunit 2	38	<0.05
P82979	SAP domain-containing ribonucleoprotein	24	<0.05
P14625	Endoplasmin	92	<0.05
P26038	Moesin	68	<0.05
Q96CT7	Coiled-coil domain-containing protein 124	26	<0.05
Q9BY44	Eukaryotic translation initiation factor 2A	65	<0.05

**Table 6:** Protein list from the mRFP, mRFP + EGF, and IP3-NLS + EGF groups

Accession number	Protein name	Molecular weight kDa	*p* value
A6NHR9	Structural maintenance of chromosomes flexible hinge domain-containing protein 1	226	<0.05
O00159	Unconventional myosin-Ic	122	<0.05
O00571	ATP-dependent RNA helicase DDX3X	73	<0.05
O43143	ATP-dependent RNA helicase DHX15	91	<0.05
O43390	Heterogeneous nuclear ribonucleoprotein R	71	<0.05
O43684	Mitotic checkpoint protein BUB3	37	<0.05
O43707	Alpha-actinin-4	105	<0.05
O60264	SWI/SNF-related matrix-associated actin-dependent regulator of chromatin subfamily A member 5	122	<0.05
O60506	Heterogeneous nuclear ribonucleoprotein Q	70	<0.05
O76021	Ribosomal L1 domain-containing protein 1	55	<0.05
O95793	Double-stranded RNA-binding protein Staufen homolog 1	63	<0.05
P02751	Fibronectin	272	<0.05
P06748	Nucleophosmin	33	<0.05
P08670	Vimentin	54	<0.05
P09651	Heterogeneous nuclear ribonucleoprotein A1	39	<0.05
P11940	Polyadenylate-binding protein 1	71	<0.05
P12814	Alpha-actinin-1	103	<0.05
P14866	Heterogeneous nuclear ribonucleoprotein L	64	<0.05
P15880	Small ribosomal subunit protein uS5	31	<0.05
P17844	Probable ATP-dependent RNA helicase DDX5	69	<0.05
P18124	Large ribosomal subunit protein uL30	29	<0.05
P19338	Nucleolin	77	<0.05
P22087	rRNA 2′-O-methyltransferase fibrillarin	34	<0.05
P22626	Heterogeneous nuclear ribonucleoproteins A2/B1	37	<0.05
P26599	Polypyrimidine tract-binding protein 1	60	<0.05
P31942	Heterogeneous nuclear ribonucleoprotein H3	37	<0.05
P31943	Heterogeneous nuclear ribonucleoprotein H	49	<0.05
P36578	Large ribosomal subunit protein uL4	48	<0.05
P39023	Large ribosomal subunit protein uL3	46	<0.05
P43243	Matrin-3	95	<0.05
P46087	Probable 28S rRNA (cytosine(4447)-C(5))-methyltransferase	89	<0.05
P46781	Small ribosomal subunit protein uS4	23	<0.05
P46940	Ras GTPase-activating-like protein IQGAP1	189	<0.05
P48681	Nestin	177	<0.05
P51991	Heterogeneous nuclear ribonucleoprotein A3	40	<0.05
P52597	Heterogeneous nuclear ribonucleoprotein F	46	<0.05
P55265	Double-stranded RNA-specific adenosine deaminase	136	<0.05
P61254	Large ribosomal subunit protein uL24	17	<0.05
P61978	Heterogeneous nuclear ribonucleoprotein K	51	<0.05
P62241	Small ribosomal subunit protein eS8	24	<0.05
P62277	Small ribosomal subunit protein uS15	17	<0.05
P62280	Small ribosomal subunit protein uS17	18	<0.05
P62753	Small ribosomal subunit protein eS6	29	<0.05
P62917	Large ribosomal subunit protein uL2	28	<0.05
P78527	DNA-dependent protein kinase catalytic subunit	469	<0.05
Q00610	Clathrin heavy chain 1	192	<0.05
Q01780	Exosome complex component 10	101	<0.05
Q02878	Large ribosomal subunit protein eL6	33	<0.05
Q03252	Lamin-B2	70	<0.05
Q05682	Caldesmon	93	<0.05
Q07020	Large ribosomal subunit protein eL18	32	<0.05
Q07065	Cytoskeleton-associated protein 4	66	<.05
Q07666	KH domain-containing, RNA-binding, signal transduction-associated protein 1	48	<0.05
Q08211	ATP-dependent RNA helicase A	141	<0.05
Q12905	Interleukin enhancer-binding factor 2	43	<0.05
Q12906	Interleukin enhancer-binding factor 3	95	<0.05
Q13813	Spectrin alpha chain, nonerythrocytic 1	285	<0.05
Q14103	Heterogeneous nuclear ribonucleoprotein D0	38	<0.05
Q14315	Filamin-C	291	<0.05
Q14980	Nuclear mitotic apparatus protein 1	238	<0.05
Q15149	Plectin	532	<0.05
Q15717	ELAV-like protein 1	36	<0.05
Q5BKZ1	DBIRD complex subunit ZNF326	66	<0.05
Q6P2Q9	Pre-mRNA-processing-splicing factor 8	274	<0.05
Q8IY81	pre-rRNA 2′-O-ribose RNA methyltransferase FTSJ3	97	<0.05
Q92841	Probable ATP-dependent RNA helicase DDX17	80	<0.05
Q96E39	RNA binding motif protein, X-linked-like-1	42	<0.05
Q96KR1	Zinc finger RNA-binding protein	117	<0.05
Q96PK6	RNA-binding protein 14	69	<0.05
Q9NR30	Nucleolar RNA helicase 2	87	<0.05
Q9NUQ6	SPATS2-like protein	62	<0.05
Q9NXF1	Testis-expressed protein 10	106	<0.05
Q9Y2W1	Thyroid hormone receptor–associated protein 3	109	<0.05
Q9Y3I0	RNA-splicing ligase RtcB homolog	55	<0.05

### Nuclear IP3 regulates chromatin association

**Figure 7:**
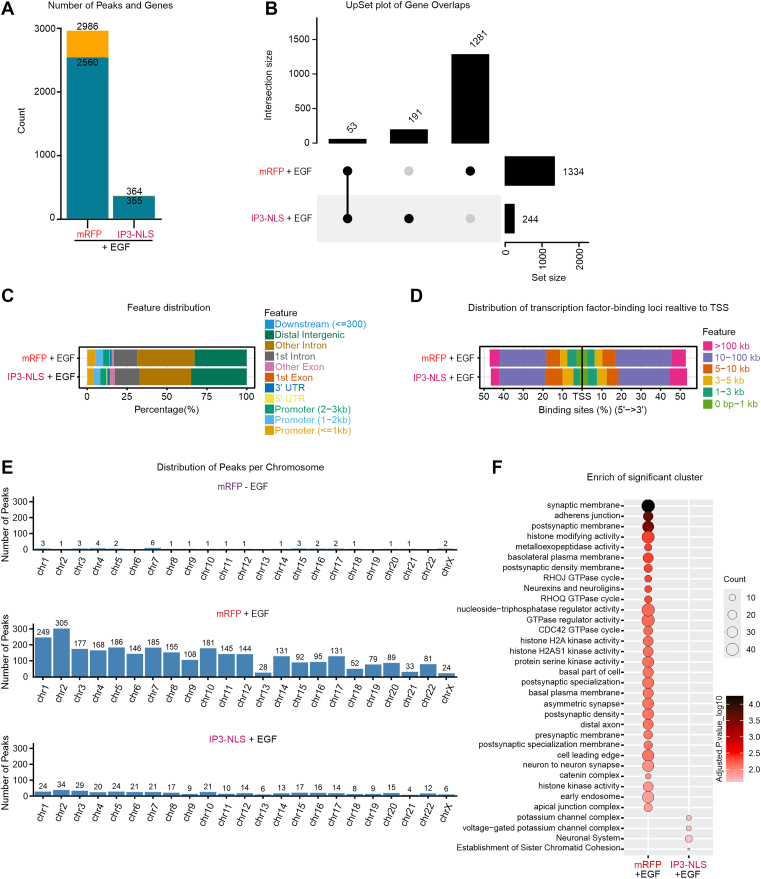
**IP3 in the nucleus drives MYH9–chromatin interactions.** ChIP-Seq was used to characterize MYH9–chromatin interactions in EGF-stimulated U2OS cells expressing either a control adenovirus with mRFP alone or IP3-NLS. *A*, quantification of MYH9 ChIP-Seq peaks and associated genes. Buffering nuclear IP3 led to a substantial reduction in MYH9 chromatin binding, with 87.8% fewer peaks and 86.1% fewer associated genes. *B*, UpSet plot illustrating gene set intersections between conditions. A total of 1281 genes were identified in the mRFP control group, but only 191 genes remained after IP3 buffering, reflecting an 85.1% decrease. *C*, genomic feature distribution of MYH9-bound loci. *D*, localization of transcription factor–binding sites relative to transcription start sites (TSS). *E*, MYH9 peaks are distributed along nearly all chromosomes. *F*, functional enrichment analysis of MYH9-bound clusters revealed significant depletion of a number of pathways, especially those related to synaptic signaling, cytoskeletal regulation, and chromatin remodeling, following nuclear buffering of IP3.

To understand how the ITPR3–nucleoskeleton complex physically influences the genome and RNA metabolism, its IP3/Ca^2+^-dependent component was investigated. Specifically, because ITPR3 acts as a nuclear Ca^2+^ release channel, while MYH9 is a Ca^2+^-sensitive nonmuscle myosin within the nucleoskeleton, we investigated whether the IP3/Ca^2+^-dependent association of MYH9 with chromatin could be the link to chromatin organization. ChIP-Seq was performed, and MYH9 was targeted to directly investigate the IP3/Ca^2+^-regulated genomic component of the complex. MYH9 ChIP-Seq experiments were conducted in EGF-stimulated cells expressing either a control adenovirus (mRFP) or with the IP3-NLS. Consistent with the hypothesis of IP3/Ca^2+^-dependent regulation, buffering nuclear IP3 decreased the number of MYH9 peaks and genes with chromatin by 87.8% and 86.1%, respectively (Fig. 7*A*). Intersections between the two sets were visualized using an UpSet plot where it was possible to observe that there were 1281 specific genes in the mRPF control group but only 191 specific genes after buffering nuclear IP3, a reduction of 85.1% (Fig. 7*B*). Further analysis of the MYH9 ChIP-Seq data revealed distinct genomic distribution patterns between conditions. Feature annotation of MYH9-bound loci showed that in mRFP + EGF, peaks were predominantly located within promoter regions (≤1 kb), first introns, and distal intergenic regions. Cells expressing IP3-NLS exhibited similar peak density across these regulatory elements (Fig. 7*C*). The distribution of transcription factor–binding loci relative to the transcription start sites (TSS) were similar in the two groups as well (Fig. 7*D*). Chromosomal mapping of MYH9 peaks demonstrated a broad distribution across all chromosomes in mRFP + EGF. This pattern was substantially diminished in IP3-NLS–expressing cells, indicating a global reduction in MYH9 binding upon nuclear IP3 buffering. Notably, analysis of nonstimulated cells revealed essentially no detectable MYH9 peaks under baseline conditions, confirming that the genomic association of this complex is not a constitutive feature but a stimulus-induced event (Fig. 7*E*). Enrichment analysis of MYH9 pathways highlighted functional categories related to cytoskeletal organization, synaptic signaling, and chromatin remodeling, each of which were notably depleted in the IP3-NLS condition (Fig. 7*F*). These findings support a model in which EGF-induced nuclear IP3/Ca^2+^ signaling acts as a trigger for the dynamic and transient reorganization of the MYH9 complex on the chromatin. Our data highlights a mechanism by which nuclear IP3 transients drive the genomic redistribution necessary for growth factor–responsive gene expression and chromatin organization.

## Discussion

The architectural regulation of the nucleus and its subsequent impact on gene expression mediated by mechanical forces is an emerging area of research. Previous studies have shown that activating G protein–coupled receptors generates cytoplasmic Ca^2+^ transients, which in turn trigger the assembly of nuclear actin filaments and influence chromatin dynamics (16). However, whether direct, autonomous nuclear IP3-mediated Ca^2+^ signaling is required for the formation of nuclear F-actin, a key element of chromatin regulation, remains unclear. It is well established that the nucleus contains the molecular machinery necessary for independent Ca^2+^ signaling, allowing nuclear Ca^2+^ levels to be regulated separately from those of the cytosol (6, 7, 8, 20).

Upon ligand activation, such as by EGF, HGF, or insulin, RTKs translocate into the nucleus, initiating IP3-mediated Ca^2+^ signals that are specific to the nuclear environment (8, 9). Given the intranuclear presence of all the machinery to generate IP3 and nuclear Ca^2+^ in response to RTK agonists, we investigated whether ligands such as EGF that increase IP3 in the nucleus are sufficient to promote the formation of nuclear actin filaments (20). This suggests a direct link between RTK activation, specific nuclear IP3/Ca^2+^ dynamics, and the reorganization of the nucleoskeleton, which may regulate gene expression and other essential nuclear functions.

The nuclear envelope consists of inner and outer lipid bilayers that can form invaginations known as the NR (6). The primary mechanism for nuclear Ca^2+^ release involves the activation of ITPRs localized within the NR (20). Consistent with this, our results show that EGF stimulation induces IP3-mediated Ca^2+^ release specifically within the nucleus, supporting the established link between EGF and nuclear Ca^2+^ signaling. Several PLC isoforms (PLCβ1, PLCγ1, PLCδ1, and PLCδ4) contribute to this process in the nucleus (21). In one such pathway, EGF-induced EGFR translocation activates intranuclear phospholipase Cδ4 (PLCD4), which generates a localized IP3 signal (5). While this sequence confirms the autonomous nature of nuclear Ca^2+^ signaling (20), the final mechanical effector connecting this signal to chromatin dynamics remains unclear.

The current findings address this knowledge gap by identifying the downstream effector that links autonomous IP3/Ca^2+^ signaling to chromatin mechanics. The data demonstrates that nuclear IP3-mediated signaling contributes to the stimulus-dependent regulation of nuclear actin assembly. While other factors, such as the nuclear concentration of G-actin and additional signaling pathways, are fundamental for actin homeostasis, our results using the nuclear-localized IP3 buffer confirm that the specific recruitment and polymerization of nuclear actin occur in response to EGF-induced nuclear IP3 transients. This conclusion is supported by the use of a selective IP3-NLS buffer, which confirmed compartment specificity, as buffering nuclear IP3 markedly diminished nuclear Ca^2+^ transients and the assembly of nuclear actin filaments. Notably, while nuclear IP3 buffering abolishes this response, nuclear F-actin assembly is preserved and appears prolonged when using the cytosolic IP3 buffer. This persistence suggests that cells continue to generate autonomous nuclear IP3 in response to EGF. The apparent prolonged nature of these filaments may reflect the unique kinetics of nuclear calcium signaling. As previously reported (8), nuclear calcium transients can exhibit slower and more sustained profiles compared to the rapid responses typically elicited by G protein–coupled agonists in the cytosol. Additionally, the mechanical components involved were defined. The data, including proteomic analysis, Western blotting, and immunofluorescence, confirmed the nuclear expression and direct physical interaction between ITPR3, MYH9, and β-actin. Bioinformatic analyses (STRING) further corroborated the interactions within the MYH9 and β-actin axis. The role of MYH9 was particularly interesting because it was found to contribute to nuclear β-actin assembly in a Ca^2+^-dependent manner *via* ITPR3, establishing it as an important link between Ca^2+^ signaling and nucleoskeletal changes. The observation that visible nuclear actin filaments occur in approximately 20% of the cells at the peak of stimulation reflects the highly dynamic nature of this process. Such polymerization events are likely restricted to specific signaling microdomains, contingent upon the spatial proximity of calcium-binding proteins and actin machinery to localized clusters of ITPR3. In these hubs, the local calcium concentration reaches the threshold required to trigger filament assembly, which may be transiently captured only when these signaling events align with the temporal and spatial resolution of confocal imaging. The dynamic nature of this complex is evidenced by the increased β-actin–MYH9 nuclear interaction following EGF stimulation, suggesting a rapid signal-dependent mechanism for complex formation and stabilization. The dynamic regulation observed in the ITPR3–MYH9–β-actin complex offers critical insights into the underlying activation mechanism. Notably, the EGF-induced enhancement of MYH9 association with ITPR3, followed by a subsequent shift toward increased MYH9–β-actin interaction, indicates a sequential activation process. It is proposed that the initial interaction between ITPR3 and MYH9 serves as a pivotal molecular positioning event. By anchoring the Ca^2+^ channel in close proximity to the MYH9–β-actin components, ITPR3 facilitates a localized release of Ca^2+^ mediated by IP3, precisely at the site of the emerging nucleoskeletal complex. This localized Ca^2+^ spike is optimally situated to initiate the Ca^2+^-dependent activation of MYH9, thereby promoting the conformational changes necessary for its integration into the functional nucleoskeleton and the subsequent remodeling of chromatin.

To establish a mechanistic link between architectural dynamics and gene function, transcriptional output was evaluated. Global RNA synthesis analysis using Click-EU revealed significant functional alterations in overall transcription rates following modulation of the ITPR3 axis. In parallel, ChIP-seq analysis successfully identified specific genomic loci targeted by this Ca^2+^-dependent mechano-signaling pathway. Collectively, these findings provide direct molecular evidence that the ITPR3–MYH9–β-actin axis functions not solely as a structural scaffold but as a rapid and dynamic regulator of chromatin architecture and gene expression. This conclusion is supported by existing literature concerning the individual components of the pathway. In particular, the role MYH9–β-actin complex aligns with its established function as a core element of the RNA polymerase II transcriptional machinery. Previous studies have demonstrated that β-actin is essential for transcription initiation, its recruitment to promoter regions, and the *in vitro* formation of pre-initiation complexes (22). Further investigations have shown that nuclear actin is required for inducible transcription by promoting the clustering of RNA polymerase II, thereby facilitating the formation of transcription factories (23).

Moreover, nuclear actin contributes to the elongation phase of transcription by associating with actively transcribed genes through direct interaction with Cdk9 of the P-TEFb complex (24). Recent evidence also links dynamic nuclear actin architecture to chromatin accessibility, demonstrating that pharmacological modulation of actin branching, *via* CK666 or cytochalasin D, induces distinct changes in chromatin landscape, as measured by ATAC-seq and alterations in histone marks (H3K9me3 and H3K27me3) (25). The nuclear role of MYH9 in transcriptional regulation has likewise been independently validated. For instance, in gastric cancer cells, nuclear MYH9 has been shown to bind directly to the CTNNB1 promoter *via* its DNA-binding domain and to interact with β-actin and RNA polymerase II, thereby promoting transcription and conferring resistance to anoikis (18).

Taken together, these results delineate a key regulatory paradigm: the ITPR3-mediated Ca^2+^ signal activates the MYH9 motor protein, which in turn orchestrates or remodels the nuclear β-actin framework. This process governs both chromatin accessibility and the dynamic organization of the transcriptional machinery. Consequently, this mechanism enables efficient recruitment, assembly, and elongation of the β-actin–dependent transcriptional complex at specific genomic loci, positioning MYH9 as a Ca^2+^-dependent effector that couples growth factor signaling to the core transcriptional apparatus *via* the dynamic nuclear actin cytoskeleton and its influence on chromatin structure. In summary, the present study provides evidence that autonomous intranuclear IP3 signaling is functionally linked to a mechanical output axis comprising ITPR3, MYH9, and β-actin. This mechanism demonstrates that local nuclear IP3 signaling rapidly controls nuclear actin assembly to modulate chromatin dynamics, offering new therapeutic targets for processes governed by nuclear Ca^2+^ signaling.

**Figure 8:**
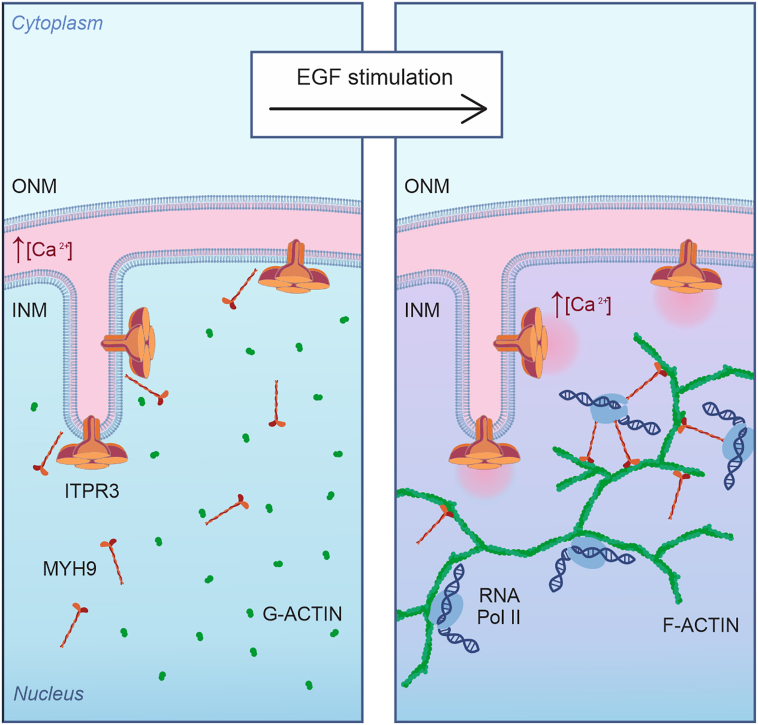
**Schematic representation of nucleoskeleton regulation upon EGF stimulation.** In the resting state, ITPR3 is associated with MYH9 within the nucleus, while actin remains in its monomeric form (G-actin). Upon EGF stimulation, the generation of nuclear IP3 activates ITPR3, triggering a localized calcium release. This calcium flux enables MYH9 to recruit and promote the assembly of nuclear actin filaments (F-actin), thereby remodeling chromatin architecture and facilitating the regulation of gene transcription.

This study identifies a fundamental, previously unrecognized mechanism by which RTK signaling rapidly and autonomously regulates nuclear actin assembly. The data demonstrated that the core of this pathway is nuclear-specific IP3/Ca^2+^ signaling, which acts as a direct trigger for nucleoskeletal reorganization. The findings establish a novel signaling and mechanical axis comprising the Ca^2+^ release channel (ITPR3), Ca^2+^-dependent protein MYH9, and actin, all of which are expressed and physically interact within the nucleus. Specifically, growth factor stimulation, such as that by EGF, initiates IP3 production in the nucleus, leading to Ca^2+^ release *via* ITPR3. This localized event drives the rapid and transient assembly of the nuclear actin–myosin nucleoskeleton (Fig. 8). The dynamic nature of this complex, as evidenced by its signal-dependent regulation, positions it as a mechanical effector connecting signaling to downstream nuclear events. Proteomic and genomic evidence together demonstrated that this mechano-signaling pathway is not merely a structural change but also a functional regulator of gene expression, targeting specific genomic loci. The transient assembly of the actin-myosin machinery, which is associated with the components of the gene transcription apparatus, suggests a mechanism by which the nucleoskeleton actively repositions genes to the transcription machinery in response to extracellular stimuli. This observation closes a major knowledge gap in nuclear biology by identifying the immediate IP3-dependent pathway that controls nuclear actin formation, offering a new paradigm for how the cell nucleus translates external growth signals into rapid, localized changes in chromatin organization and gene transcription.

## Experimental procedures

### Cell culture

U2OS (ATCC, HTB-96), NIH/3T3 (ATCC, CRL-1658), and MDCK.2 (ATCC, CRL-2936) cell lines were obtained from the American Type Culture Collection, and their authenticity was confirmed by short tandem repeat profiling. Cells were cultured in high-glucose DMEM (Gibco) supplemented with 10% (v/v) FBS (Gibco), 100 units/ml penicillin, and 100 μg/ml streptomycin and incubated at 37 °C in a humidified atmosphere containing 95% O_2_ and 5% CO_2_ (17). *Mycoplasma* contamination was tested using the MycoAlert Detection Kit (Cat. #LT07-318, Lonza).

### Immunoprecipitations

Cells were lysed in ice-cold buffer (20 mM Hepes, pH 7.0, 10 mM KCl, 2 mM MgCl_2_, 0.5% Nonidet P-40, and 1% protease and phosphatase cocktail inhibitors (Cat. #78445, Thermo Fisher Scientific)). Lysates were vortexed for 1 min, incubated on ice for 10 min, and centrifuged at 1500*g* for 5 min at 4 °C to pellet the nuclei. The supernatant was centrifuged at 16,000*g* for 20 min at 4 °C to obtain the non-nuclear fraction. The nuclear pellet was washed three times with lysis buffer, resuspended in NETN buffer (150 mM NaCl, 1 mM EDTA, 20 mM Tris–HCl, pH 8.0, 0.5% Nonidet P-40), and sonicated for 1 min. Nuclear lysates were collected after centrifugation at 16,000*g* for 20 min at 4 °C.

For immunoprecipitations (IPs), 400 μg of protein lysate was resuspended in 500 μl of IP buffer containing 1% Triton X-100, 150 mM NaCl, 10 mM Tris-HCl pH 7.5, 0.5% NP-40, 10% sucrose, and protease and phosphatase inhibitors at 4 °C. Then, 5 to 10 μg of antibodies were added to the non-nuclear and nuclear fractions for 1 h at 4 °C with rotation. Protein A/G magnetic agarose beads (Cat. #78609; Thermo Fisher Scientific) was added to each sample. The protein lysate and beads were incubated at 4 °C under rotary agitation for 4 h. The beads were pelleted using a magnetic stand and washed with PBS for 5 times (17).

For the IPs of ITPR3 in MDCK cells, the non-nuclear and nuclear fractions were collected, and 400 μg of lysates were incubated with 5 μg of ITPR3 antibody for 1 h at 4 °C. For the IP of β -actin in U2OS, the cells were treated with 200 nM Lat-B for 12 h or one group was left untreated as controls. Nuclear and non-nuclear fractions were prepared as described above for the MDCK cells. The nuclear lysate was incubated with 10 μg of anti-β-actin antibody for 1 h at 4 °C with rotation. For the IPs of ITPR3 and MYH9 in the nuclear fractions of U2OS. The nuclear fraction collected, and 400 μg of lysates were incubated with 5 μg of ITPR3 or MYH9 antibodies for 1 h at 4 °C. For the IP of ITPR3 in U2OS, cells expressing mRFP or mRFP-IP3-NLS were treated and untreated with EGF (200 ng/ml) for 60 s. The nuclear fraction was collected, and 400 μg of lysate was resuspended in IP buffer containing 1% Triton X-100, 150 mM NaCl, 10 mM Tris-HCl (pH 7.5), 0.5% NP-40, 10% sucrose, and protease inhibitors. The mixture was incubated with 5 μg of ITPR3 antibody for 1 h at 4°C with rotation. Protein A/G magnetic agarose beads were added to each sample, and the resulting protein-bead complexes were incubated for 4 h at 4 °C with rotary agitation. The beads were isolated using a magnetic stand, washed five times with PBS, and the collected samples were submitted for mass spectrometry analysis.

### Mass spectrometry

Mass spectrometry (MS) was performed at Yale's Keck Laboratory, as described previously (17). IPs were collected, fractionated by SDS-PAGE, and visualized with Coomassie blue (Cat. #B8647; Sigma Aldrich). The gel bands were cut and submitted for Gel Protein ID proteomic analysis. The products were digested and fractionated using a high-performance hybrid Quadrupole-Orbitrap LC-MS/MS system (Thermo Scientific Q Exactive Plus). For peptide identification, the data were analyzed using Proteome Discoverer (version 2.5) (Thermo Fisher Scientific) software and searched in-house using the Mascot algorithm (version 2.8.3) (Matrix Science). The data were searched against the Swissprotein database (v2025_02) with taxonomy restricted to *Homo sapiens* (20,422 sequences), a database of common mass spectrometry contaminants (304 sequences) or against database restricted to Canis familiaris, and a custom database containing the sequences of the constructs used in the experiment. Peptide identifications were accepted if they could be established with a probability greater than 95.0%. Protein identifications were accepted if they could be established with greater than 99.0% probability (assigned by the Protein Prophet algorithm) and contained at least two identified peptides. Proteins containing similar peptides that could not be differentiated based on MS/MS analysis alone were grouped to satisfy the principles of parsimony. Proteins with significant peptide evidence were grouped into clusters. MS data were analyzed using Scaffold v4.0 or higher (Proteome Software Inc.) to validate MS/MS-based peptide and protein identifications.

### Bioinformatic analysis of the ITPR3 interactome

Interactions among ITPR3, MYH9, and β-actin were analyzed using the STRING (26) (https://string-db.org/) database. STRING analysis, performed with a 97% interaction confidence threshold, supported the potential nuclear interactions identified in our mass spectrometry data.

### Western blot

Cells were lysed in ice-cold buffer (20 mM Hepes, pH 7.0, 10 mM KCl, 2 mM MgCl_2_, 0.5% Nonidet P-40, protease and phosphatase inhibitors). Lysates were vortexed for 1 min, incubated on ice for 10 min, and centrifuged at 1500*g* for 5 min at 4 °C to pellet nuclei. The supernatant was further centrifuged at 16,000*g* for 20 min at 4 °C to obtain the non-nuclear fraction. The nuclear pellet was washed three times with lysis buffer, resuspended in NETN buffer (150 mM NaCl, 1 mM EDTA, 20 mM Tris–HCl, pH 8.0, 0.5% Nonidet P-40), and sonicated for 1 min. Nuclear lysates were collected after centrifugation at 16,000*g* for 20 min at 4 °C. For immunoblotting, 40 μg of protein lysates were mixed with sample buffer, heated at 95 °C for 5 min, and separated on 4 to 10% gradient SDS-PAGE gels (Bio-Rad). Proteins were transferred to polyvinylidene fluoride membranes, blocked in 5% nonfat milk in TBST (20 mM Tris–HCl pH 7.6, 150 mM NaCl, 0.1% Tween-20), and incubated with primary antibodies overnight at 4 °C. After washing, the membranes were incubated with HRP-conjugated secondary antibodies for 1 h and developed using SuperSignal West Pico (Thermo Fisher Scientific) (5). Subsequently, the films were scanned and analyzed using ImageJ software. Results were normalized to the percentage of nuclear or non-nuclear fractions, using the more abundant fraction as a reference.

### Immunofluorescence

Immunofluorescence assays were performed to evaluate the expression and localization of ITPR3, MYH9, and β-actin in MDCK.2, NIH/3T3, and U2OS cells. Cells were fixed in ice-cold methanol or 4% paraformaldehyde and blocked with 1% bovine serum albumin in PBS for 1 h. The cells were then incubated overnight at 4 °C with primary antibodies against ITPR3, MYH9, and β-actin, diluted in blocking buffer. Detection was performed using Alexa Fluor–conjugated secondary antibodies: Alexa Fluor 647 for ITPR3, 546 for MYH9, and 488 for β-actin (Molecular Probes). Secondary antibody incubation was performed for 1 h at room temperature, protected from light. Nuclei were counterstained with Hoechst 33342 (Molecular Probes), and the samples were washed with PBS before mounting with ProLong Gold Antifade Reagent (Thermo Fisher Scientific). Imaging was conducted at 63× magnification (NA 1.4) using a GSD/TIRF HP microscope (Leica Microsystems) or Leica SP8 Gated STED 3X super-resolution microscope. Contact point analyses were performed along the Z-axis using the LAS X software with volume rendering. All images were processed using Adobe Photoshop CS6 (Adobe Systems) (5).

### Antibodies

The primary antibodies used for Western blot were at the indicated dilutions: anti-ITPR3 (1:500; BD-Biosciences, 610313), anti-MYH9 (1:1000; Invitrogen, Pas-17025), anti-Lamin B1 (1:5000; Abcam, Ab16048), anti-α-tubulin (1:10,000; Sigma-Aldrich, T6199), and anti-β-actin (1:10,000; Sigma, A5441). The secondary antibodies used were as follows: HRP-conjugated anti-mouse (1:10,000; Invitrogen, A28177) and anti-rabbit (1:5000; Cell Signaling, A27036).

The primary antibodies used in immunofluorescence were at the indicated dilutions: anti-ITPR3 (1:100; BD-Biosciences, 610313), anti-MYH9 (1:100; Invitrogen, PA5-17025), and β-actin–conjugated Alexa Fluor 488 (1:1000; Invitrogen, MA1-140-A488). The secondary antibodies used were Alexa Fluor anti-rabbit 546 (1:1000; Invitrogen) and Alexa Fluor anti-mouse 647 (1:1000; Invitrogen). The nuclear probe was Hoechst 33342 (1:10,000; Life Technologies).

The primary antibodies used in IPs and ChIP-seq were anti-ITPR3 (5 μg; BD-Biosciences 610313), anti-MYH9 (5 μg; Abcam, Ab238131), and anti-β-actin (10 μg; Sigma, A5441).

The specificity of β-actin, ITPR3, and MYH9 antibodies were confirmed by mass spectrometry. Furthermore, ITPR3 and MYH9 antibodies were validated by knockdown or knockout experiments.

### Generation of stable cell lines

For the generation of stable U2OS and NIH/3T3 cells expressing the Nuclear Actin Chromobody (nAC-TagGFP2; Chromotek) construct, cells were plated at a density of 2 × 10^5^ per well in 6-well plates and incubated overnight at 37 °C. Transfection was performed in OPTI-MEM medium using Lipofectamine 2000 or 3000 and 100 ng of DNA from the nAC-TagGFP2 construct, according to the manufacturer's instructions (Invitrogen). The cells were incubated in OPTI-MEM medium for 4 h at 37 °C in a 5% CO_2_ atmosphere. Subsequently, the medium was replaced with basal medium (DMEM) containing 1 mg/ml G418 for cellular selection.

### ITPR3 knockdown

Three independent siRNA duplexes targeting the human ITPR3 gene (GenBank ID:3710) were utilized for transient knockdown experiments to avoid sequence-specific off-target effects. A nontargeting scrambled siRNA (scramble) with no known homology to human genes was used as the negative control. All oligonucleotides were obtained as 21 bp HPLC-purified duplexes (Integrated DNA Technologies) and resuspended in RNase-free water to a stock concentration of 100 μM (see Table 4). U2OS-nAC and NIH/3T3-nAC stable cell lines were seeded in 6-well plates at a density adjusted to reach 60 to 70% confluence on the day of transfection. The working concentration for siRNA was empirically optimized to 40 nM, minimizing cytotoxicity while ensuring maximal silencing efficiency. For complex formation, the siRNA stock was diluted in OPTI-MEM–reduced serum medium (Gibco). The transfection reagent (Lipofectamine 2000, Invitrogen) was diluted in OPTI-MEM. The two solutions were combined, mixed gently by pipetting, and incubated for 20 min at room temperature. The siRNA–lipid complexes were added dropwise to the culture medium. Cells were then incubated at 37 °C in 5% CO_2_ for 48 h. The silencing efficiency was verified using a Western blot assay (17).

### Visualization of nuclear actin filaments

The U2OS-nAC and NIH/3T3-nAC stable cell lines were plated on sterile glass coverslips at a density of 2 × 10^5^ cells per well and cultured overnight at 37 °C. For cell starvation and synchronization, the standard culture medium was replaced with serum-free DMEM, and the cells were incubated for an additional 12 h. Immediately following the specific stimulation duration, cells were fixed by submerging the coverslips in ice-cold methanol for 10 min. Nuclei were counterstained with Hoechst 33342 (1 μg/ml for 10 min), and images were acquired using a Leica GSD/TIRF HP or Leica SP8 Gated STED 3X super-resolution microscope. Image processing and subsequent analysis were conducted using LAS X software (Leica Microsystems) and ImageJ (16). Cells were counted as “positive” for nuclear F-actin if they exhibited at least two distinct, linear filament structures within the nuclear boundary that were not present in the prestimulation frame.

### Adenoviral infection

Cells were seeded on coverslips at a density of 2 × 10^5^ and cultured overnight at 37 °C in complete DMEM. The cells were then transduced with adenoviruses encoding mRFP, mRFP-IP3-NLS, and mRFP-IP3-NES at 100 PFU per well and incubated for 48 h. The medium was replaced with serum-free DMEM for 12 h before Ca^2+^ imaging and time-course experiments. Adenovirus was produced at Viralquest Inc or Avantor sciences.

### Calcium imaging

U2OS cells were plated and cultured in 35 mm dishes (Corning). The cells were transduced with adenoviral constructs to express the mRFP-IP3-NLS and mRFP-IP3-NES, as well as mRFP alone, which served as a control. Following transduction, cells were maintained in serum-free medium 24 h prior to the experiment. To monitor intracellular Ca^2+^ dynamics, the cells were loaded with 5 μM Fluo-4 AM (Invitrogen) for 22 min at 37 °C, 48 h after transduction. The coverslips were placed in a custom-made perfusion chamber. Excess dye was removed by washing twice with Hepes buffer (130 mM NaCl, 5 mM KCl, 1 mM MgSO_4_·7H_2_O, 1.2 mM KH_2_PO_4_, 1.25 mM CaCl_2_·2H_2_O), and the cells were kept in Hepes during imaging. Ca^2+^ signaling was stimulated with 200 ng/ml of EGF. Fluorescence was recorded using Zeiss LSM 880 or Bruker Opterra II swept-field confocal microscopes and quantified using Fiji (ImageJ) software. The duration of Ca^2+^ changes within the regions of interest was calculated as the average across each regions of interest, and the results were expressed as F/F_0_ × 100, where F_0_ represents the baseline fluorescence intensity (27).

### Detection of global RNA synthesis

Nascent RNA was detected using the Click-iT RNA Alexa Fluor 488 Imaging Kit (Click-EU), following the manufacturer's instructions (Thermo Fisher Scientific, C10329). U2OS cells were seeded in 96-well plates at a density of 2 × 10^4^ cells per well in DMEM supplemented with 10% FBS. The cells were infected with adenoviruses encoding mRFP, mRFP-IP3-NLS, or mRFP-IP3-NES at 100 PFU per well and incubated for 48 h. The medium was then replaced with FBS-free DMEM for 24 h before stimulation with EGF (200 ng/ml). The second treatment was performed with EGF for 6 h under the same conditions. For EU incorporation, the cells were incubated for 1 h with prewarmed DMEM containing 100 μM EU and EGF. After labeling, the cells were rinsed with PBS and fixed with 4% paraformaldehyde for 15 min at room temperature (RT), followed by permeabilization with 0.5% Triton X-100 for 15 min at RT. EU incorporation was detected using 100 μl of Click-iT reaction cocktail (Click-iT RNA reaction buffer, CuSO_4_, Alexa Fluor 488 azide, and Click-iT buffer additive) for 90 min at RT. The cells were then washed with rinse buffer, followed by PBS, and counterstained with Hoechst for 15 min. Finally, the cells were washed with PBS and imaged.

### ChIP–seq

ChIP was performed using the Pierce Magnetic ChIP Kit (Thermo Scientific, 26157), following the manufacturer’s instructions. Briefly, U2OS cells were seeded at a density of 4 × 10^5^ cells/ml in 60 mm single dishes and cultured for 24 h. Prior to treatment, the basal medium was replaced with serum-free medium. The following day, cells were treated with 200 ng/ml EGF for 60 s. Untreated cells served as the control group. Cells were then fixed with 1% formaldehyde in DMEM at room temperature for 5 min, quenched with DMEM containing 200 mM glycine for 5 min at room temperature, and washed with ice-cold PBS supplemented with 1% Halt Cocktail (Thermo Fisher Scientific). Fixed cells underwent lysis and MNase digestion according to the kit protocol. Chromatin was sheared using a FB120 sonicator with CL-18 tip (Thermo Fisher Scientific), applying six cycles of 30-s sonication with 30-s intervals at 25% amplitude. The lysate was cleared by centrifugation at 9000*g* for 5 min at 4 °C. The supernatant was diluted with 410 μl of IP dilution buffer and incubated overnight at 4 °C with rotation, using 20 μl of ChIP-Grade Protein A/G Magnetic Beads (Thermo Fisher Scientific) prebound to 10 μg of rabbit anti–non-muscle myosin IIA monoclonal antibody (Abcam). Immune complexes were washed five times with IP Wash Buffer 1 (provided in the kit), followed by a single wash with IP Wash Buffer containing 5 M sodium chloride. DNA was eluted using ChIP Elution Buffer (Thermo Fisher Scientific), incubated at 65 °C for 40 min, and purified using the DNA Clean-Up Column (Thermo Fisher Scientific). ChIP–seq libraries were prepared using the DNA SMART ChIP-Seq Kit (Takara Bio, 634865), following the manufacturer’s instructions. Sequencing was performed using paired-end 150 bp reads on an Illumina NovaSeq X system at the Genomics Core of the Yale Stem Cell Center (YCGA).

### ChIP-seq data processing and peak calling

Raw paired-end ChIP-seq reads were first subjected to quality control using FastQC (v0.11.9) (28) to assess sequencing quality metrics. Adapter sequences and low-quality bases were trimmed using Trim Galore (v0.6.7), applying a 3-bp 5′ end clip for Read1 and a 15-bp 5′ end clip for Read2, followed by merging of the multiple sequencing files per sample. Trimmed reads were then aligned to the human reference genome (hg38) using Bowtie2 (v2.4.2) (29). For paired-end reads, the maximum insert size was set to 2000 bp. Alignments with mapping quality <30 and reads mapping to mitochondrial chromosomes were excluded. Resulting BAM files were sorted and indexed using SAMtools (v1.16). To remove potential artifacts arising from off-target amplification inherent to the SMART ChIP-seq library preparation method, the aligned BAM files were processed with SMARTcleaner (30). SMARTcleaner identifies and removes reads derived from false amplification of poly(T/A) genomic sequences based on strand-specific patterns, generating a cleaned BAM file with significantly reduced background noise. Cleaned BAM files were subsequently re-indexed and used for downstream analyses. Quality assessment of aligned and cleaned reads was performed using deepTools plotFingerprint to visualize signal enrichment and library complexity across ChIP and input samples. Narrow peaks were called using MACS2 (v2.2.7.1) (31) with the SPMR option for fragment pileup normalization and genome size parameters appropriate to each species. Input control samples were included when available to improve peak specificity. Overall, this workflow integrates standard ChIP-seq data preprocessing, SMART-specific artifact removal, high-confidence peak calling, annotation, and motif discovery to ensure reliable identification of protein–DNA interactions in SMART-based ChIP-seq datasets.

### Peak annotation and functional enrichment analyses

All downstream bioinformatic analyses were performed in R (v4.4.2). ChIP-seq peaks generated by MACS2 were filtered to retain only those with a fold enrichment greater than 5 and a –log10(q-value) exceeding 2, ensuring the inclusion of high-confidence binding regions. Peak annotation was performed using the ChIPseeker package (v1.42.1) (32), with the human genome assembly hg38 provided by the TxDb.Hsapiens.UCSC.hg38.knownGene package (v3.20.0) as reference and gene annotation obtained from the org.Hs.eg.db package (v3.20.0). Each peak was assigned to its corresponding genomic region (promoter, exon, intron, intergenic, *etc.*) and associated to the nearest TSS within ±3 kb. The feature distribution and transcription factor–binding loci relative to the TSS were visualized using the plotAnnoBar and plotDistToTSS functions from the ChIPseeker package. Visualizations were generated using ggplot2 package (v3.5.2) (33), including bar plots to compare overall peak numbers across samples and to display their chromosomal distribution. Gene set intersections among ChIP-seq samples were assessed using UpSet plots from ComplexHeatmap package (v2.25.1) (34). Genes associated with significant peaks were extracted and analyzed for functional enrichment using clusterProfiler (v4.14.6) (35) and ReactomePA (v1.50.0) (36). Enrichment analyses included KEGG pathways, Gene Ontology (GO: Biological Process, Molecular Function, and Cellular Component), and Reactome databases. Statistical significance was assessed using the Benjamini–Hochberg correction for multiple testing, retaining terms with adjusted *p*-value (*p*. adjust) < 0.05. Results were visualized as dot plots showing the top enriched biological categories for each condition.

### Statistical analysis and reproducibility

All experiments were performed with at least three independent biological replicates, unless otherwise specified in the figure’s legends. Quantitative contact point analyses were performed in five cells per treatment condition. Statistical tests were selected based on the data normality. For normally distributed data, unpaired two-tailed Student’s *t*-tests (mean ± SD) or one-way ANOVA with Dunnett’s multiple comparisons test were used. Statistical analyses were performed using GraphPad Prism 10. Relative peptide abundance in the nuclear and non-nuclear fractions was assessed, and proteins with the highest specificity and sensitivity were identified using Student’s *t* test with a significance threshold of *p* < 0.01.

### Data availability

Data analyzed in this study will be available by the corresponding author upon reasonable request.

The ChIP-seq data generated for this study have been deposited in the National Center for Biotechnology Information (NCBI) Gene Expression Omnibus (GEO) database and are accessible through GEO Series accession number GSE324124 (https://www.ncbi.nlm.nih.gov/geo/query/acc.cgi?acc=GSE324124). The scripts were deposited on Zenodo (https://doi.org/10.5281/zenodo.18926308).

The mass spectrometry proteomics data have been deposited to the ProteomeXchange Consortium *via* the PRIDE partner repository with the dataset identifiers PXD075764, PXD075767, PXD075677, and PXD075787.

## Supporting information

This article contains supporting information.

## Conflict of interest

The authors declare that they have no conflicts of interest with the contents of this article.
