# Supplementary material for: Regulation of chromatin dynamics by a calcium-dependent nucleoskeleton
Source: J Biol Chem. 2026 May 6;302(6):113104. doi: 10.1016/j.jbc.2026.113104 (PMC13254598; doi:10.1016/j.jbc.2026.113104)
Supplement: Figures S1-S6 [file mmc1.docx]

Supporting Information

**Regulation of chromatin dynamics by a calcium-dependent nucleoskeleton**

María José González^1,2, #^, Michele Angela Rodrigues^1,2, #^, Santo Diprima^2,4^, Dejian Zhao^3^, Thais Fernandes Bassani^1^, Clara Couto Fernandez^1^, Emma Kruglov^2^, Michael H. Nathanson^2^, and Dawidson Assis Gomes^1,*^

^1^Department of Biochemistry and Immunology, Universidade Federal de Minas Gerais (UFMG), Av. Antônio Carlos, 6627, Belo Horizonte – MG, 31270-901, Brazil.

^2^Section of Digestive Diseases, Internal Medicine, Yale University CT, New Haven, USA 333 Cedar St, New Haven, CT, 06520-8056, USA.

^3^Yale Center for Genome Analysis, Department of Genetics, Yale School of Medicine, New Haven, CT 06510, USA.

^4^Center for Omics Sciences, IRCCS San Raffaele Scientific Institute, Milan, Italy.

^#^Contributed equally

*Correspondence: dawidson.gomes@gmail.com

ORCID: 0000-0001-7714-991X

Figure S1

Figure S2

Figure S3

Figure S4

Figure S5

Figure S6

Video S1

**
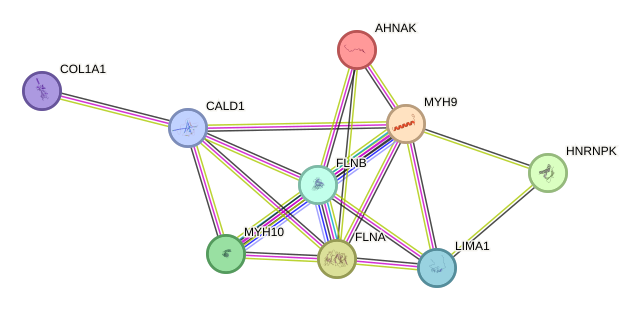
**

Figure S1. **Interactome of the nine proteins found in the mass spectroscopy analysis after β-actin (ACTB) IP in untreated cells with latrunculin.** STRING analysis of the nine proteins listed in Table 2, as shown in the Venn diagram of Fig. 1*F* and Volcano plot of Fig.1*G*.


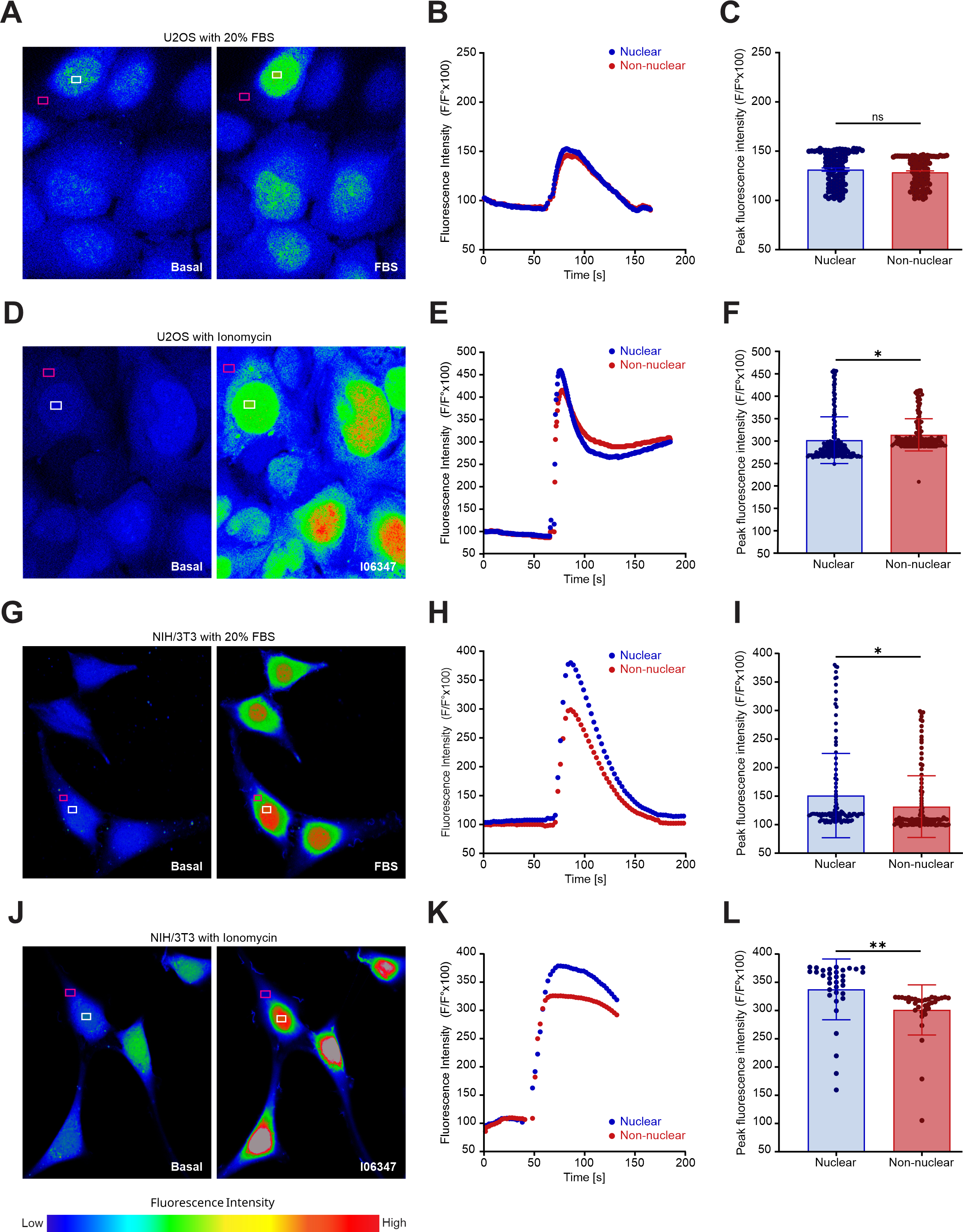


Figure S2. **Stimulation with FBS or ionomycin increases nucleoplasmic calcium.** *A*, Images were taken before (basal) and after stimulation with 20% FBS in U2OS cells. *B*, Graph showing calcium dynamics after stimulation with FBS. *C*, Quantification of peak calcium intensity in the nucleus and cytoplasm (non-nuclear). *D*, Images were taken before (basal) and after stimulation with 2 µM ionomycin in U2OS cells. *E*, Graph showing calcium dynamics after stimulation with ionomycin. *F*, Quantification of peak calcium intensity in the nucleus and cytoplasm. *G*, Images were taken before (basal) and after stimulation with 20% FBS in NIH/3T3 cells. *H*, Graph showing calcium dynamics after stimulation with FBS. *I*, Quantification of peak calcium intensity in the nucleus and cytoplasm. *J*, Images were taken before (basal) and after stimulation with 2 µM of ionomycin in NIH/3T3 cells. *K*, Graph showing calcium dynamics after stimulation with ionomycin. *L*, Quantification of peak calcium intensity in the nucleus and cytoplasm. Scale bars = 10 μm. (n = 3 independent experiments). The p value was obtained by unpaired two-tailed t-test (**p* < 0.05 and ***p* < 0.01. ns = not significant). Data are means ± SD. Images were acquired using an LSM 880 confocal microscope.


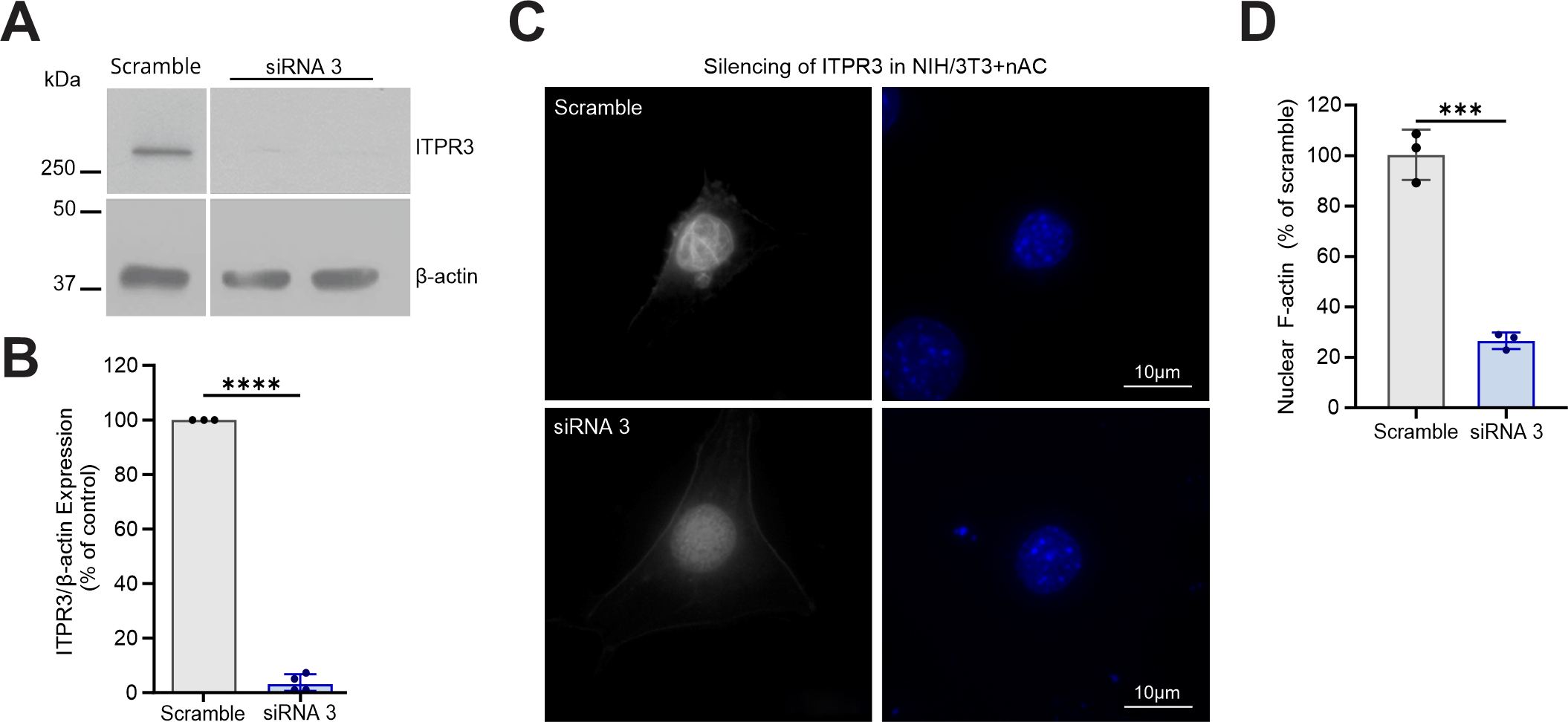


Figure S3. **ITPR3 knockdown reduces F-actin formation in NHI/3T3 cells.** *A*, Representative western blot showing the knockdown of ITPR3 in NIH/3T3 cells transfected with siRNA sequence 3 (siRNA3). *B*, Quantification of ITPR3 expression after knockdown with siRNA3. Scramble siRNA was used as a control. ITPR3 expression was reduced by 96.91 ± 5% using siRNA3. Data are presented as the mean ± SD. (n = 3). Statistical analysis was performed using an unpaired two-tailed t-test (*****p* ≤ 0.0001). *C*, Representative images showing reduction in nuclear F-actin formation after ITPR3 knockdown (bottom). *D*, Graph shows the percentage of cells with nuclear F-actin after ITPR3 knockdown in cells stimulated with 20% FBS. The percentage of cells with nuclear F-actin was reduced after silencing ITPR3 with siRNA3 compared with scramble. n = 3. Statistical analysis was performed using an unpaired two-tailed t-test (****p* ≤ 0.001).

**
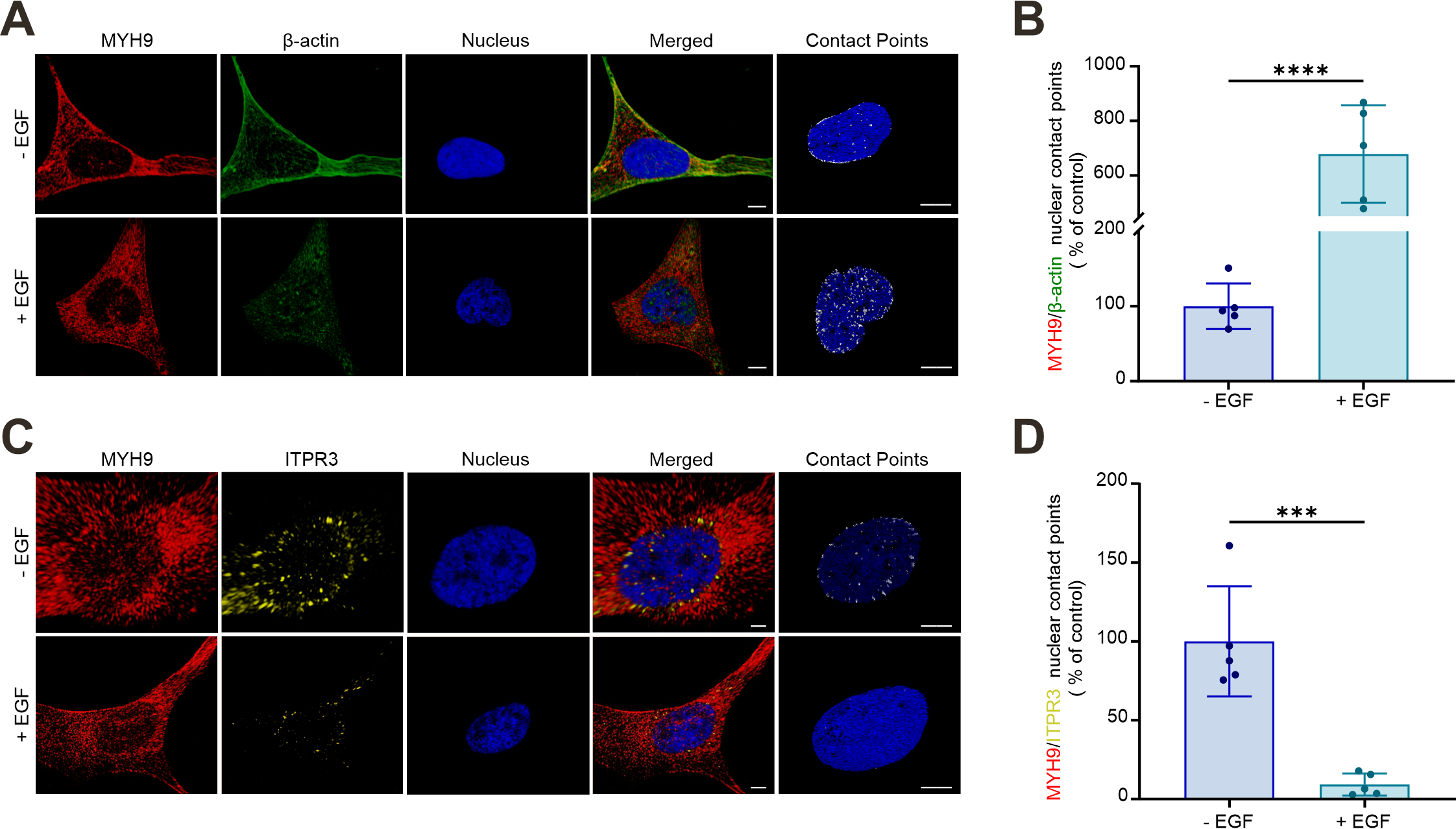
**

Figure S4. **EGF increases co-localization of MYH9 and β-actin while decreasing co-localization of MYH9 and ITPR3 in the nucleus.** *A*, Representative images of U2OS cells without stimulation (top) and after stimulation with 200 ng/mL EGF (bottom). MYH9 (red), β-actin (green), nuclei (blue), merged images, and co-localization of MYH9 and β-actin within the nucleus (white dots) are shown (left to right). *B*, Quantitative analysis of immunofluorescence data showing an increase in MYH9–β-actin contact points in the nucleus after EGF stimulation. Statistical analysis was performed using an unpaired two-tailed t-test (*****p* ≤ 0.0001). *C*, Representative images of U2OS cells without stimulation (top) and stimulated with 200 ng/mL EGF (bottom). MYH9 (red), ITPR3 (yellow), nuclei (blue), merged images and nuclear contact points (white dots) are shown (left to right). *D*, Quantitative analysis of immunofluorescence shows a decrease in nuclear MYH9–ITPR3 contact points after EGF stimulation. Scale bars: 10 μm. Data represents mean+SD from three independent experiments. Statistical analysis was performed using an unpaired two-tailed t-test (****p* ≤ 0.001). Images were acquired using a Leica gated STED super-resolution microscope.

**
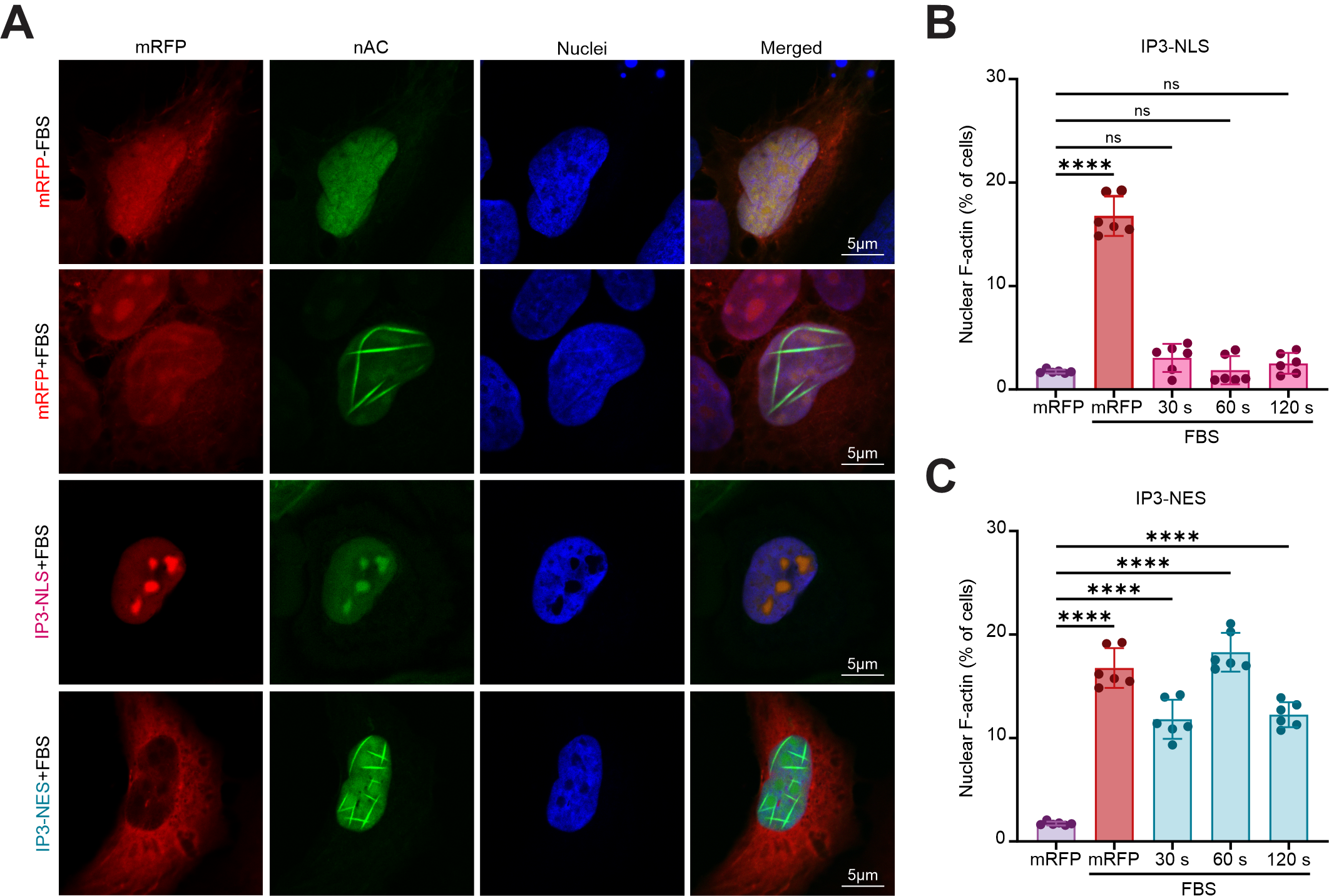
**

Figure S5. **An increase in nuclear IP3 is necessary for assembly of nuclear actin filaments.** *A*, Representative images of U2OS stably expressing nAC after infection with adenovirus to express mRFP, IP3-NLS or IP3-NES buffers, and stimulation with 20% FBS for 30 s, 60 s and 120 s. Scale bars = 2 μm, 5 μm or 10 μm. *B*, Buffering IP3 in the cytoplasm (with IP3-NES) does not block FBS-induced actin filament formation in the nucleus while, *C*, buffering IP3 in the nucleus, with IP3-NLS, does block actin filament formation in the nucleus. Percentage of cells that formed nuclear actin filaments after expression in IP3-NLS and IP3-NES buffers after stimulation with 20% FBS. mRFP - EGF was used as a negative control, and mRFP + EGF was used as a positive control. For each experiment, the percentage of positive cells with F-Actin were counted per replicate (representative quantification from 3 independent experiments). Statistical analysis was performed using two-way ANOVA with Dunnet correction (*****p* ≤ 0.0001 or ns = not significant). Data are means ± SD. Images were acquired using a Leica gated STED super-resolution microscope.

**
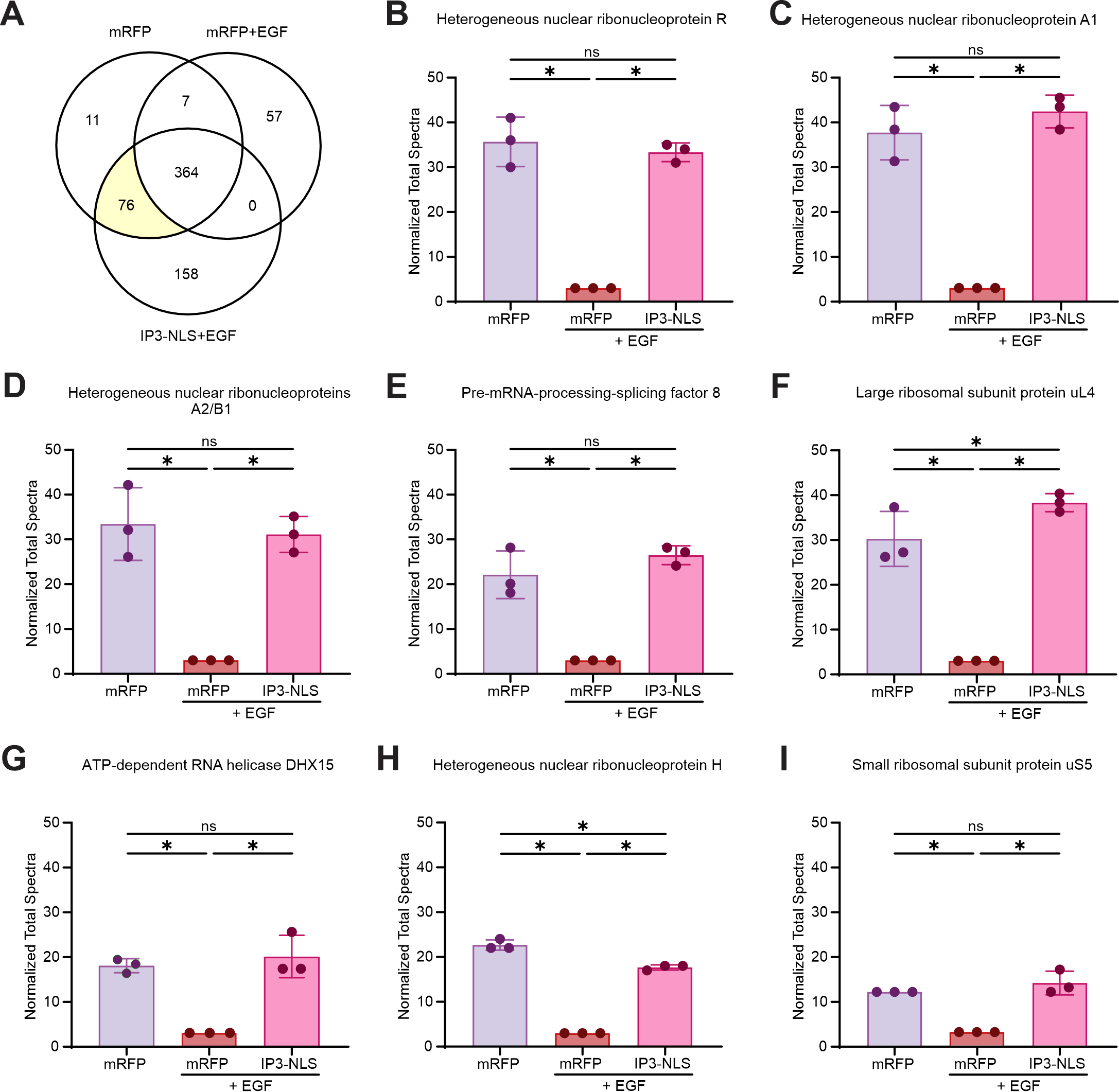
**

Figure S6. **Nuclear IP3 buffer inhibits EGF-induced alterations in ITPR3-associated proteins linked to RNA metabolism.** *A*, Venn diagram illustrates the overlap of proteins identified by mass spectrometry in U2OS cells with mRFP, mRFP + EGF, and IP3-NLS + EGF. The yellow-shaded region represents 76 proteins found in the mRFP and IP3-NLS + EGF groups. *B* to *I*, Normalized total spectra of representative proteins from the mRFP, mRFP + EGF and IP3-NLS + EGF groups. These include: *B*, Heterogeneous nuclear ribonucleoprotein R; *C*, Heterogeneous nuclear ribonucleoprotein A1; *D*, Heterogeneous nuclear ribonucleoproteins A2/B1; *E*, Pre-mRNA-processing-splicing factor 8; *F*, Large ribosomal subunit protein uL4; *G*, ATP-dependent RNA helicase DHX15; *H*, Heterogeneous nuclear ribonucleoprotein H, and; *I*, Small ribosomal subunit protein uS5. Data are presented as mean ± SD; **p* < 0.05, One-way ANOVA corrected for multiple comparisons (Benjamini, Krieger, and Yekutieli method)**;** (n = 3). The minimum value for the lower-expressed proteins was three peptides.

**Video S1:** U2OS cells expressing the nAC construct were first monitored after stimulation with FBS or ionomycin.
